# Artificial miRNAs targeting CAG repeat expansion in ORFs cause rapid deadenylation and translation inhibition of mutant transcripts

**DOI:** 10.1007/s00018-020-03596-7

**Published:** 2020-07-21

**Authors:** Adam Ciesiolka, Anna Stroynowska-Czerwinska, Paweł Joachimiak, Agata Ciolak, Emilia Kozlowska, Michal Michalak, Magdalena Dabrowska, Marta Olejniczak, Katarzyna D. Raczynska, Dominika Zielinska, Magdalena Wozna-Wysocka, Wlodzimierz J. Krzyzosiak, Agnieszka Fiszer

**Affiliations:** 1 Department of Molecular Biomedicine, Institute of Bioorganic Chemistry, Polish Academy of Sciences, Noskowskiego 12/14, Poznan, Poland; 2 Laboratory of Structural Biology, International Institute of Molecular and Cell Biology, Ks. Trojdena 4, Warszawa, Poland; 3 Department of Genome Engineering, Institute of Bioorganic Chemistry, Polish Academy of Sciences, Noskowskiego 12/14, Poznan, Poland; 4 Department of Gene Expression, Institute of Molecular Biology and Biotechnology, Adam Mickiewicz University in Poznan, Wieniawskiego 1, Poznan, Poland; 5 Center for Advanced Technology, Adam Mickiewicz University, Wieniawskiego 1, Poznan, Poland

**Keywords:** miRNA, CAG repeats, Polyglutamine diseases, Huntington’s disease, Translational inhibition

## Abstract

Polyglutamine (polyQ) diseases are incurable neurological disorders caused by CAG repeat expansion in the open reading frames (ORFs) of specific genes. This type of mutation in the *HTT* gene is responsible for Huntington’s disease (HD). CAG repeat-targeting artificial miRNAs (art-miRNAs) were shown as attractive therapeutic approach for polyQ disorders as they caused allele-selective decrease in the level of mutant proteins. Here, using polyQ disease models, we aimed to demonstrate how miRNA-based gene expression regulation is dependent on target sequence features. We show that the silencing efficiency and selectivity of art-miRNAs is influenced by the localization of the CAG repeat tract within transcript and the specific sequence context. Furthermore, we aimed to reveal the events leading to downregulation of mutant polyQ proteins and found very rapid activation of translational repression and *HTT* transcript deadenylation. Slicer-activity of AGO2 was dispensable in this process, as determined in *AGO2* knockout cells generated with CRISPR-Cas9 technology. We also showed highly allele-selective downregulation of huntingtin in human HD neural progenitors (NPs). Taken together, art-miRNA activity may serve as a model of the cooperative activity and targeting of ORF regions by endogenous miRNAs.

**Electronic supplementary material:**

The online version of this article (10.1007/s00018-020-03596-7) contains supplementary material, which is available to authorized users.

## Introduction

Non-coding RNAs (ncRNAs) are a large, diverse group of transcripts that do not contain information about protein sequence but mainly play a crucial role in the post-transcriptional regulation of gene expression. Examples of ncRNAs are short interfering RNAs (siRNAs) and microRNAs (miRNAs), which constitute a large family of short (~21 nt) RNAs [1–4]. SiRNAs activate RNA-induced silencing complex (RISC) to carry out the AGO2-mediated cleavage of a transcript within a perfectly matched siRNA-mRNA duplex, followed by mRNA degradation [5]. In contrast, the miRNA strand guides the miRNA-induced silencing complex (miRISC) to interact with only partially complementary sequences within the transcripts in animal cells, causing translational inhibition and mRNA transcript decay following deadenylation [6–10].

Functional miRNA-binding sites are usually localized within the 3′ untranslated region (UTR) but might also be present within the open reading frame (ORF) [11–15] and 5′UTR [16–18]. These latter sites are considered as less functional than those in the 3′UTR as miRISCs cannot avoid collision with the scanning small ribosomal subunit and rapidly translocating ribosomes [19]. Importantly, the efficiency of the miRNA-mediated regulation of gene expression may depend on the number of miRNA-binding sites within the regulated target [20] and the distance between these sites [21]. The more target sites at an optimal distance on mRNA there are, the higher the observed inhibitory effect is, caused by cooperative interaction between miRISCs bound to neighboring sites [22].

**Fig. 1:**
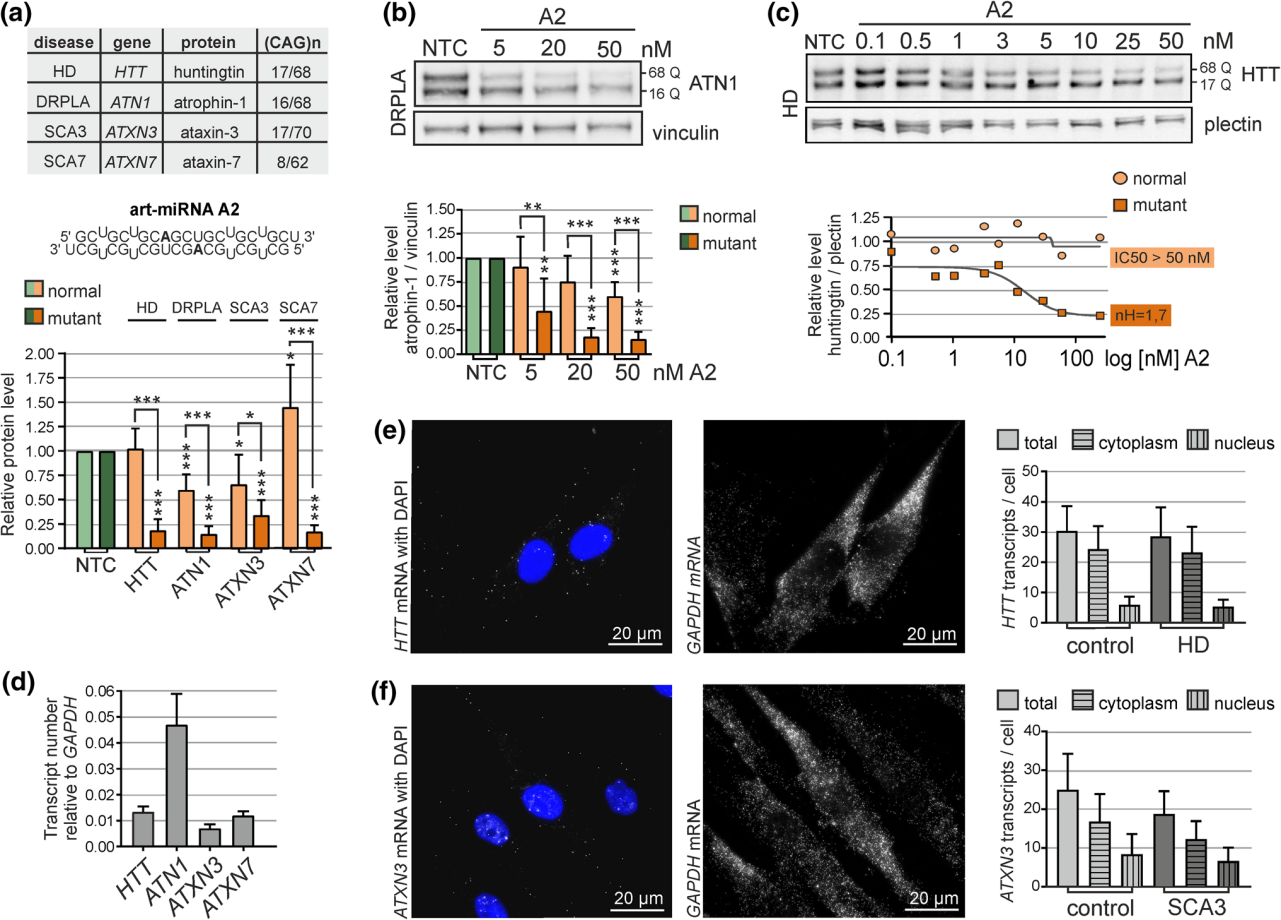
A2 activity in patient-derived fibroblasts and the characteristics of targeted transcripts. **a** Upper panel: table with information about investigated models of polyQ diseases. In the last column CAG repeat tract lengths (normal/mutant allele), present in fibroblasts cells used, are given. Middle panel: sequence of art-miRNA A2 and predicted base-pairing of two strands within a duplex. Lower panel: results compiled from the western blot analysis showing HTT, ATN1, ATXN3 or ATXN7 protein levels in HD, DRPLA, SCA3 or SCA7-patient-derived fibroblasts, respectively, after transfection with 50 nM A2. Vinculin, GAPDH and plectin were used as reference proteins. NTC—cells treated with non-targeting siRNA. The results of HTT and ATXN7 downregulation are from published studies [33, 39]. The following statistical tests were used: one-sample *t* test with a hypothetical value = 1 for allele expression level; unpaired *t* test with Welch's correction for comparisons of normal and mutant allele expression. *n* = 3 **b** Western blot analysis of atrophin-1 levels in DRPLA-patient-derived fibroblasts lysed 48 h after transfection with 5, 20 or 50 nM A2. NTC—cells treated with non-targeting siRNA. Data were analyzed using one-way ANOVA (Bonferroni multiple comparisons test). *n* = 3. **c** Western blot analysis of huntingtin levels in HD fibroblasts lysed 72 h after transfection with the indicated concentration of A2. The results are presented as dose–response curves that were used to calculate the indicated Hill coefficient. NTC—cells treated with non-targeting siRNA. See Figure S1A for more data. *n* = 3 **d** Non-allele-specific quantification of *HTT*, *ATN1*, *ATXN3* and *ATXN7* transcripts with ddPCR. The results were obtained from two sets of cDNA from independent cultures of each of five fibroblast cell lines. See Figure S1C for more data. **e**, **f** Representative smFISH images for *HTT* (E) and *ATXN3* (**f**) mRNAs in HD patient- and SCA3 patient-derived fibroblasts, respectively. DAPI was used for nuclear staining. Middle panels: *GAPDH* transcripts detected in the same cells. Right panels: non-allele-specific quantification of *HTT* and *ATXN3* signals in healthy and patient-derived fibroblasts. Signals were counted from at least 50 cells. NTC used in experiments presented in this figure was BlockIT siRNA

SiRNAs and miRNAs, as negative regulators of gene expression, are often used in the development of therapeutic approaches. One of the examples are strategies for incurable and progressive neurodegenerative polyglutamine (polyQ) diseases which include Huntington’s disease (HD), spinal bulbar muscular atrophy (SBMA), dentatorubral–pallidoluysian atrophy (DRPLA) or spinocerebellar ataxia (SCA) types 1, 2, 3, 6, 7 and 17 (Fig. 1a). These disorders are caused by the expansion of CAG repeat sequences within the ORFs of specific genes, so that the normal alleles contain 10–20 CAG repeats, whereas mutant alleles usually 40–70 CAG repeats. Due to a location of mutation within ORF, mutated gene encode protein with an expanded polyQ tract [23, 24].

One promising therapeutic approach is the elimination of mutant gene expression by directly targeting the mutation site in the transcript, i.e., the expanded CAG repeat tract [25, 26]. In a series of studies from David Corey’s and our groups, the effects of particular oligonucleotides, hereafter called CAG repeat-targeting artificial miRNAs (art-miRNAs), were tested in various polyQ disease models [27–40] (Table S1). The common feature of these oligonucleotides is the presence of specific mismatches in the interaction with the targeted CAG repeat tract, making these oligonucleotides similar to miRNAs. Allele-selective downregulation of mutant polyQ proteins by art-miRNAs most probably results from preferential activation of the silencing mechanism when multiple miRISCs are present on the expanded repeat tract. The targeted transcript level was less affected than the protein level as art-miRNAs did not induce the substantial mRNA cleavage typical of siRNAs [27, 28, 30]. Moreover, a study of the mechanism of action of art-miRNAs suggested cooperative silencing by miRISCs located on the expanded repeat tract, as revealed by dose-response experiments, and the involvement of AGO2 and GW182, as shown by RNA immunoprecipitation and siRNA-based knockdown experiments [30]. Intriguingly, the activities of art-miRNAs in various polyQ disease models differed significantly, and minor differences in oligonucleotide sequence largely affected the observed activity (Table S1). Therefore, we decided to investigate the details of the activated silencing process in the context of further development of this approach and as an example of miRNA-based targeting of ORF regions.

In this study, we aimed to elucidate the key factors affecting silencing efficiency of art-miRNAs and determine the mechanism of their action. For this purpose we used cells with endogenous mutant gene expression, including human neural progenitors (NPs), as well as we constructed several dedicated cellular models. We compiled the results of testing our most effective art-miRNA, A2 [33], to highlight the variance in its activity in different polyQ disease models. We show that allele-selectivity of art-miRNAs is determined by the localization of CAG repeat tract in ORF and strengthened by specific sequence of huntingtin (*HTT*) transcript. Moreover, we demonstrate that A2 induced rapid mRNA deadenylation and translation inhibition and AGO2 was not required in activated silencing mechanism.

## Materials and methods

### Cell lines

HEK 293T (American Type Culture Collection) and host Flp-In T-REx-293 cell lines (Thermo Fisher Scientific) were cultivated in Dulbecco’s Modified Eagle’s Medium (Sigma-Aldrich), containing 10% fetal bovine serum (Biowest), penicillin-streptomycin solution (Sigma-Aldrich), 2 mM l-glutamine (Sigma-Aldrich). Additionally, for 16CAG and 98CAG Flp-In T-REx-293 cell culture 100 µg/ml hygromycin B (Thermo Fisher Scientific) and 5 µg/ml blasticidin S (Thermo Fisher Scientific) was supplemented. Patient-derived fibroblasts (Coriell Institute, SCA3 GM06153: 17/70 CAG repeats in *ATXN3*; HD GM04281: 17/68 CAG repeats in *HTT*, DRPLA GM13716: 16/68 CAG repeats in *ATN1*, SCA7 GM03561: 8/62 CAG repeats in *ATXN7*; and control line GM05565) were grown in Eagle’s Minimal Essential Medium (Sigma-Aldrich) supplemented with 10% fetal bovine serum (Sigma-Aldrich), antibiotic–antimycotic solution (Sigma-Aldrich), 2 mM GlutaMAX (Gibco) and MEM non-essential amino acids (Sigma-Aldrich).

Human neural progenitors (NPs) were derived from HD induced pluripotent stem cells (iPSC) ND42222 (19/109 CAG repeats in *HTT*) obtained from NINDS Human Genetics Resource Center (Coriell Institute). For neural induction STEMdiff SMADi Neural Induction Kit (STEMCELL Technologies) was used according to monolayer protocol, following manufacturer's instructions. Briefly, iPSC were grown in Essential 8 (Gibco) medium on Geltrex (Gibco) coated 6-well plate until 70–80% confluence was reached. Then, iPSCs were dissociated to single cells by incubation with 0.5 mM EDTA in PBS for 10 min. Cells were counted using TC20 Automated Cell Counter (Bio-Rad) and resuspended at 1 × 10^6^ cells/ml density for seeding in STEMdiff Neural Induction Medium with SMADi and 10 nM Y-27632 (all from STEMCELL Technologies). For further cultivation cells were detached using Accutase (STEMCELL Technologies) and after third passage they were grown in STEMdiff Neural Progenitors Medium (STEMCELL Technologies). Expression of *SOX1*, *SOX2*, *PAX6,* and *NES* markers was confirmed by ICC (Supplementary Figure S4A) and by RT-qPCR (Supplementary Figure S4B). All cell lines were cultured at an appropriate cell confluence at 37 °C in 5% CO_2_. Cell banks were stored in liquid nitrogen.

### RNA oligonucleotides

All siRNA oligonucleotides (Table S2) were synthesized by Metabion or Future Synthesis, dissolved in water to 100 µM concentration and stored at − 80 °C. To obtain 20 µM duplexes sense and antisense strands were diluted in annealing buffer, heated for 1 min in 90 °C and kept for gradual cooling at room temperature for 45 min.

### Transfection

Lipofectamine 2000 (Invitrogen) was used to transfect HEK 293T, Flp-In T-REx-293 cells and fibroblasts with plasmids and oligonucleotides, accordingly to the manufacturer’s protocol. 24 h prior to transfection cells were plated after estimation of cell number. To optimize and monitor transfection efficiency control fluorescent BlockIT siRNA (Invitrogen) or control plasmid encoding GFP (System Biosciences) was used. Cells were harvested at specific time points indicated in figure legends. Briefly, HEK 293T line (120,000 cells/well seeded into 24-well plate) was co-transfected with 100 ng of plasmid of pmirGLO construct and 50 nM oligonucleotide using 1.5 µl Lipofectamine 2000 in 300 µl medium. Generated Flp-In T-REx-293 lines (160,000 cells/well seeded into 12-well plate) were transfected with 100 nM oligonucleotide using 4 µl Lipofectamine 2000 in 1.2 ml of medium. Transfection of NPs was performed at fourth or fifth passage using 2 μl of siPORT Amine (Ambion) per well of 6-well plate in 1 ml of complete medium. After 3 h medium was replaced with fresh one and after next 24 h the medium was changed for the media lacking Y-27632. NPs were harvested using Accutase fixed 48 h post-transfection

### Protein isolation and western blot

Cells were collected at specific time points for particular experiments (which are given in Figure legends), e.g. time points selected for most efficient downregulation of specific proteins in fibroblasts and NPs. Cell pellets were washed once with PBS and lysed with PB buffer (60 mM Tris-base, 2% SDS, 10% sucrose, 2 mM PMSF). Next, the cell extract was heated in 95 °C for 5 min and protein concentration was estimated based on measurement at 280 nm using DeNovix spectrophotometer. Equal amounts (~30 µg) of total protein were diluted in loading buffer and heated in 95 °C for 5 min and run on SDS-polyacrylamide gels: 5% stacking, 10% resolving gel in Tris/glycine/SDS buffer for ataxin-3 and luciferase detection; 3–8% NuPAGE Tris acetate gels (Thermo Fisher Scientific) in XT Tricine buffer (Bio-Rad) with cooling in ice-water bath for atrophin-1 and huntingtin detection. Next, proteins were wet-transferred to nitrocellulose membrane (GE Healthcare) and specific primary (anti-ataxin-3, anti-huntingtin, anti-atrophin-1, anti-vinculin and anti-Fluc) and horseradish peroxidase-conjugated secondary antibodies (anti-rabbit or anti-mouse) were used. All antibodies used are given in Table S3**.** The immunodetection was performed using WesternBright Quantum HRP Substrate (Advansta). The chemiluminescent signals were scanned from membranes using GBOX documentation system (Syngene) and the bands were quantified using Gel-Pro Analyzer.

### RNA isolation, RT-qPCR and ddRT-PCR

After cell lysis in TRI Reagent (ThermoFisher)*,* Direct-zol RNA MiniPrep kit (ZymoResearch) or Total RNA Zol-Out kit (A&A Biotechnology) was used for total RNA isolation*.* For Flp-In T-REx-293 cell lines, a fraction of lysates prepared in Cytoplasmic Lysis Buffer [PBS, 0.1% NP40, cOmplete EDTA-free Protease Inhibitor Cocktail (Roche)] was mixed with four volumes of TRI Reagent for further isolation. The concentration of isolated total RNA was assessed by measurement at 260 nm using DeNovix spectrophotometer. Reverse transcription was performed using High-Capacity cDNA Reverse Transcription Kit (Applied Biosystems) and random hexamer primers (Promega), according to the manufacturer's protocols. RT-qPCR was performed using SsoAdvanced Universal SYBR Green Supermix (Bio-Rad) and CFX Connect Real-Time System (Bio-Rad), according to the manufacturer's protocols and established guidelines for qPCR. Digital droplet PCRs (ddPCRs) were prepared using DG8 cartridges and gaskets, QX200 Droplet Generation Oil and QX200 EvaGreen Digital PCR Supermix (BioRad) and performed on QX200 Droplet Digital PCR System (BioRad), according to the manufacturer's protocols. All primer sequences are listed in Table S4.

### Hill coefficient calculation

The obtained results of an average relative protein level have been fitted by GraphPad Prism to the Hill equation curve (*y *= *a *+ (*b *− *a*)/[1 + (*K*/*x*)^*N*^], where *x* is oligonucleotide concentration, *y* is relative protein expression, *a* is minimal value of *y*, *b* is maximal value of *y*, *K* is fitting parameters and *N* determines the slope of the curve and the value is the Hill coefficient, nH).

### smFISH

Probes, buffers and protocol from Stellaris RNA FISH technology (Biosearch Technologies) were used. Probes 3′-labelled with Quasar 670 dye were used for human *HTT* (cat # SMF-20836-5) and *ATXN3* (Custom Stellaris RNA FISH probes designed using online Stellaris probe designer, sequences are listed in Table S5), and with CAL Fluor 590 dye for *GAPDH* (cat # SMF-2026-1). Cells were fixed in 4% paraformaldehyde in PBS for 20 min at RT, then prehybridized in Wash Buffer A containing 10% formamide for 5 min at RT. Hybridization was performed in Hybridization Buffer with 10% formamide at 37 °C overnight. Washing was performed with Wash Buffer A for 30 min at 37 °C and next with Wash Buffer B for 5 min at RT. SlowFade Diamond Antifade Mountant (Thermo Fisher Scientific) was used for nuclear staining. Images were captured with Leica DMI6000 inverted fluorescence microscope equipped with DFC360 FX camera. Excitation/emission filters sets were Leica A for DAPI, Chroma 49005 and 49009 for CAL Fluor 590 and Quasar670, respectively. To visually examine data, a maximum intensity z-projection of all of slices in each stack were created using ImageJ. Signals were simplistically attributed as nuclear based on DAPI staining. Quantification of individual RNA FISH spots was done using the StarSearch software (https://www.seas.upenn.edu/~rajlab/StarSearch/launch.html).

### Luciferase-based plasmids containing CAG repeat tracts

The plasmids were generated on the basis of the pmirGLO Vector (Promega) encoding Firefly luciferase (Fluc) and Renilla luciferase (Rluc). CAG repeat tract sequences were inserted into *Fluc* gene, either downstream (“3′UTR” and “3′ORF” plasmids; between SalI and XbaI restriction sites) or upstream (“5′ORF” plasmids; between restriction sites for EcoRI and NdeI inserted in two steps using QuikChange II XL Site-Directed Mutagenesis Kit (Agilent Technologies)). “3′ORF” plasmid was generated by mutation of “3′UTR” plasmid in the *Fluc* gene STOP codon. *ATXN3* and *HTT*-specific inserts with normal and mutant CAG repeat tracts were obtained by PCR using cDNA from fibroblast cell lines (SCA3 and HD) and primers with specific restriction sites. Short synthetic inserts (containing 17 CAG repeats and including specific restriction sites) were chemically synthesized (Sigma-Aldrich) and annealed for cloning. For longer synthetic inserts we used in vitro repeat expansion method known as Synthesis of Long Iterative Polynucleotide (SLIP) [41, 42] with the “17CAG” insert as initial template for the repeat tract expansion. After obtaining first plasmid with expanded CAG tract, we further modified a protocol and used two plasmids with various lengths of CAG tract in SLIP. This approach allowed for generating longer expansion at one step. Due to DNA polymerase slipping the lengths of mutated constructs are slightly different. Ligation of inserts with plasmids was performed using T4 Ligase (Promega) according to the manufacturer's procedure. Next, competent DH5α *E. coli* cells were transformed and plasmids were isolated using Endotoxin-free MidiPrep kit (Qiagen). Due to technical problems mutant synthetic inserts are not included for the set of “5′ORF” constructs. Sequences of DNA oligonucleotides used for cloning are given in Table S6.

### Flp-In T-REx-293 cell lines for inducible and stable expression of CAG repeat tracts

All components of the designed dual-luciferase system were cloned into pcDNA5/FRT/TO vector (Invitrogen) for obtaining inducible expression in Flp-In T-REx-293 cell lines of either normal or mutant *HTT* fragment. This vector was integrated into the genome via Flp recombinase-mediated DNA recombination at the FRT site. Sequences of *Fluc* and Nano luciferase with PEST domain (*NlucP*) were cloned from pmirGLO and pNL1.2 (Promega) vectors, respectively. A bidirectional inducible promoter system (BI-16) capable of reproducible coexpression of two proteins was constructed [43]. Additional SV40pA sequences were cloned from pNL1.2 (Promega) at respective sites. The full exon 1 of *HTT* containing either 16 or 98 CAG repeats was amplified in PCR and these inserts were cloned upstream of *NlucP* sequence to obtain expression of *HTT-NlucP* fusion gene. Sequences of DNA oligonucleotides used for cloning are given in Table S7. The pcDNA5/FRT/TO-based expression constructs and pOG44 vector were co-transfected (at 1:9 ratio) into Flp-In T-REx-293 host cells using Lipofectamine 2000, according to manufacturer’s protocol. Selection of hygromycin-resistant monoclones that contain stably integrated expression cassette was performed using 100 μg/ml hygromycin B, according to manufacturer’s protocols. Additional details on the DNA cloning procedure and the generation of these stable cell lines are given in Supplementary Methods.

### Luciferase assay

**Fig. 2:**
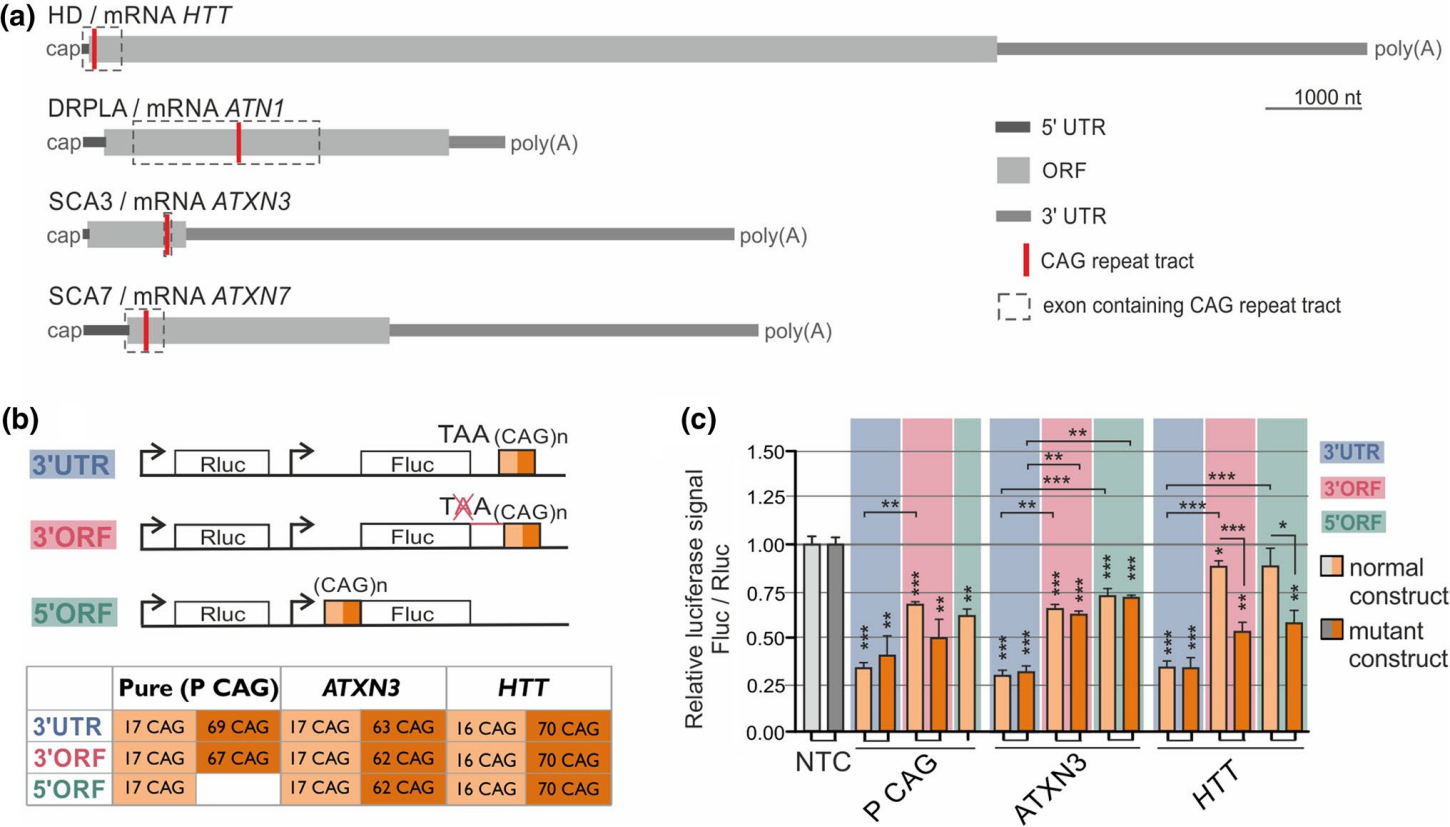
Impact of repeat tract length, location and sequence surrounding the targeted region on efficiency of downregulation by A2. **a** Scheme of *HTT* (NM_002111.8), *ATN1* (NM_001007026.2), *ATXN3* (NM_004993.6) and *ATXN*7 (NM_000333.3) transcripts with CAG repeat tract locations, UTRs and ORFs marked. **b** Scheme of dual-luciferase-based constructs containing the *Fluc* gene fused with the indicated CAG repeat tracts in various locations: the 3′UTR and 3′ and 5′ sites of the ORF. The exact lengths of the tracts in constructs containing CAG repeats without (‘Pure’) or with gene-specific surrounding sequences (from *ATXN3* or *HTT*) are given in a table. **c** Luciferase assay performed 24 h after cotransfection of HEK 293T cells with 50 nM A2 and 100 ng of the indicated plasmids. NTC—light gray: cells treated with pmirGLO plasmid and non-targeting siRNA, dark gray: “5′ORF”-modified pmirGLO plasmid treated with non-targeting siRNA (signal normalization details are given in Materials and Methods). Data were analyzed using one-way ANOVA (with Bonferroni multiple comparisons test). *n* = 3

For the results presented in the Figs. 2 and S2 assays were performed using Dual-Luciferase Reporter Assay System (Promega), accordingly to the manufacturer’s protocol. Briefly, cells were lysed 24 h after transfection in Passive Lysis Buffer (Promega), followed by the luciferase activity measurement using Centro LB 960 Luminometer (Berthold Technologies). The Fluc measurement data was normalized firstly to Rluc signal in the sample, next to Fluc/Rluc signal ratio obtained in cells co-transfected with negative control (NTC, non-targeting siRNA) and particular plasmid, and finally to Fluc/Rluc signal ratio measured for cells treated with plasmid lacking CAG repeat insert and particular oligonucleotide.

For the results presented in the Figs. 4 and 5 cells were lysed using Cytoplasmic Lysis Buffer [PBS, 0.1% NP40, cOmplete EDTA-free Protease Inhibitor Cocktail (Roche)] according to the REAP method [44]. Next, the lysates was used in the Nano-Glo Dual-Luciferase Reporter Assay System (Promega) and measured with Victor X4 Multilabel Plate Reader (Perkin Elmer), according to the manufacturer’s instructions. Background values of NlucP and Fluc signals at *t *= 0 h were subtracted. Nluc signals for A2- or siHTT-treated cells were normalized to NlucP measurement obtained at specific time points for siRluc-treated cells. Finally, NlucP signal was normalized to Fluc signal in a respective sample.

### Polysome profiling

The protocol was adapted from [45, 46]. Briefly, 5 × 10^6^ Flp-In T-REx-293 16 CAG or 98CAG cells were seeded into 55 cm^2^ plate in medium without antibiotics. After 24 h cells were transfected using selected oligonucleotides at final concentration of 100 nM. After additional 12 h *HTT* exogene expression was induced using 1 µg/ml doxycycline (Sigma-Aldrich) and after 3 h 100 µg/ml cycloheximide (Sigma-Aldrich) was added to inhibit translation elongation and fix ribosomes on transcripts. After 5 min of incubation at 37 °C cells were washed with ice-cold PBS containing cycloheximide and harvested in 1.5 ml of this buffer by scraping. Cells were collected by centrifugation at 300 rpm for 5 min at 4 °C and lysed in 500 µl ice-cold lysis buffer (10 mM HEPES pH 7,9; 1.5 mM MgCl_2_; 10 mM KCl; 0.5 mM DTT; 1% Triton X-100, 100 µg/ml cycloheximide) containing also 100 μ/ml of RNasin (Promega). After 10 min incubation on ice lysates were centrifuged at 1500*g* for 5 min at 4 °C. Supernatant was collected and OD was measured at 260 nm.

10–60% sucrose gradients were prepared using Gradient Station (BioComp) in buffer containing 100 mM KCl, 20 mM HEPES pH 7.6; 5 mM MgCl_2_, 100 µg/ml cycloheximide; 5 µ/ml RNasin and Protease Inhibitor Cocktail (Roche). 10 OD was loaded onto cooled sucrose gradients and centrifuged at 39,000 rpm for 2 h and 40 min at 4 °C using ultracentrifuge and SW 41Ti rotor (Beckman Coulter) About twenty 0.5 ml fractions were collected using Piston Gradient Fractionator (BioComp). Next, 0.5 ml of TRI-reagent (Ambion) was added to each fraction and subsequently RNA was isolated, including treatment with DNase I. Equal volumes of total RNA were reverse transcribed and *HTT-NlucP* and *GAPDH* expression levels were determined by qRT-PCR.

### Poly(A) tail length measurements

The analysis was performed based on a polyG/I extension method [47] using the Poly(A) Tail-Length Assay Kit (Thermo Fisher Scientific). In these experiments 5 µg/ml actinomycin-D (Sigma-Aldrich) was added to the medium of 16CAG and 98CAG Flp-In T-REx-293 cells to stop transcription. 200 ng of isolated RNA from selected time points were taken for poly(A) tail length analysis that was performed following manufacturer’s protocol. Specific primers used are listed in Table S4. For estimation of poly(A) tail lengths, a product obtained using gene-specific reverse primer was used as a reference. PCR products were analyzed on 2100 Bioanalyzer using DNA 1000 Kit (Agilent).

### Generation of AGO2 knockout and AGO2(D597A) mutant stable cell lines and transient AGO2 overexpression

CRISPR-Cas9-mediated AGO2 knockout and AGO2(D597A) mutant cell lines were established using previously generated Flp-In T-REx-293 98CAG cells. For AGO2 knockout Cas9_sg1 and Cas9_sg2 plasmids, encoding sgRNA1 and sgRNA2 which are specific for target sequences within exon 2 of *AGO2* gene, were used. Cas9_sg3 encoding sgRNA3, binding to sequence within exon 14, was used for AGO2(D597A) mutant cell line. To generate these plasmids, sense and antisense DNA strands of sgRNAs were annealed and ligated into pSpCas9(BB)-2A-GFP (PX458) (Addgene) plasmid, digested with the FastDigest BpiI (Thermo Fisher Scientific). Chemically competent *E. coli* GT116 cells (InvivoGen) were transformed with the plasmids, plated onto ampicillin selection plates (100 μg/ml ampicillin) and incubated overnight at 37 °C. The plasmids were isolated using the Gene JET Plasmid Miniprep kit (Thermo Fisher Scientific) and analyzed by Sanger sequencing. For nucleofection Flp-In T-REx-293 98CAG cells were electroporated with the Neon Transfection System (Invitrogen). Briefly, 1 × 10^5^ cells were harvested, resuspended in Buffer R and electroporated with 1 μg of plasmid DNA (500 ng of each Cas9_sg1 and Cas9_sg2 plasmids) in 10 μl tips using the following parameters: 1100 V, 20 ms, two pulses. For AGO2(D597A) cell line generation, cells were electroporated with 1 μg of plasmid DNA and 1 μl of 100 μM single-stranded donor oligonucleotide (ssODN) (IDT) harboring GAC to GCC codon change. Selection of clones is described in Supplementary Materials and Methods. The oligonucleotide sequences are included in Table S8. For AGO2 overexpression, AGO2 coding sequence was amplified using PCR from pIRESneo FLAG/HA Ago2 plasmid (Addgene, #10822) [48] and cloned into pcDNA3.1(+) (Invitrogen) between HindIII/BamHI sites.

### Statistical analysis

Analyses were performed using GraphPad Prism software. Two-tailed *p* value < 0.05 was considered significant and is depicted on the graphs by: *0.05 > *p *> 0.01; **0.01 > *p *> 0.001; ***p < 0.001. All experiments which resulted in statistically-analyzed quantitative data were repeated at least three times (the exact number of biological replicates, *n*, is given in figure legends). Depending on the experimental setup specific statistical tests were used and are indicated in figure legends. The error bars in the graphs represent standard deviations.

## Results

### A2-mediated silencing of different polyQ disease-related genes is varied

A large set of art-miRNAs have been tested in fibroblasts derived from patients with several polyQ diseases (Table S1). We have now complemented our results obtained with the A2 oligonucleotide [33, 38, 39] and present a direct comparison of its activity when used at the same concentration in cell lines bearing similar repeat tract lengths in mutant alleles, i.e., 62–70 CAG repeats (Fig. 1a). Overall, we observed that the efficiency and allele-selectivity of A2 art-miRNA differed in various models of polyQ diseases. The highest degree of allele-selectivity was achieved for the downregulation of *HTT* and *ATXN7*, where mutant huntingtin and ataxin-7 proteins were lowered to ~ 20% of control level, without reduction in normal protein levels. For ataxin-7 we also observed significant increase in normal protein level after A2 treatment [39]. Normal *ATN1* and *ATXN3* alleles were more susceptible to downregulation, but levels of normal atrophin-1 and ataxin-3 was decreased by no more than 50% of the control level with a relatively high concentration of A2 (50 nM) (Fig. 1a). In all of the examined disease models, A2 caused an allele-selective decrease in mutant protein levels at a wider range of concentrations used, as shown in the DRPLA model example (Fig. 1b). Mutant atrophin-1 was downregulated with 20 nM A2 to less than 20% of the control level without a reduction in normal protein level. Additionally, we analyzed the activity of A2 at a very wide range of concentrations in HD and SCA3 models to assess potential cooperative activity, as was previously reported for other art-miRNAs [30, 31]. We obtained a Hill coefficient (nH) value considerably > 1 (~ 1.7) what suggests cooperative activity of the silencing machinery for the downregulation of mutant huntingtin (Figs. 1c, S1A). On the other hand, among the models investigated, the ataxin-3 protein was decreased in the least allele-selective manner, and the obtained nHs suggest cooperative silencing of both the normal and mutant alleles by A2 (Fig. S1B). Overall, these and previous observations clearly show the targeted transcript-dependent activity of art-miRNAs.

### The cellular level of polyQ disease-related transcripts is relatively low

To characterize several polyQ disease-related transcripts in more detail, we first performed their quantification in patient-derived fibroblast cells using ddPCR. The respective transcripts were present at very low levels relative to *GAPDH,* and similar expression levels of *HTT* and *ATXN7*, slightly lower *ATXN3* levels and considerably higher *ATN1* levels were observed (Fig. 1d). Five separately analyzed fibroblast cell lines showed some variation in transcript levels but without outstanding tendency for fibroblasts with the mutation in specific gene, e.g., the *HTT* mRNA level in HD fibroblasts (Fig. S1C).

In addition, we performed single-molecule fluorescent in situ hybridization (smFISH) for the precise quantification and visualization of selected transcripts in fibroblasts. Microscopic analyses showed approximately 30 *HTT* transcripts and approximately 20 *ATXN3* transcripts per cell (Figs. 1e, f, S1D). Interestingly, the ratio of transcripts in the cytoplasm to those in the nucleus was approximately 4:1 and 2:1 for *HTT* and *ATXN3*, respectively. This suggests that larger cytoplasmic fraction of *HTT* transcripts, in comparison to *ATXN3* transcripts, is available for the activation of RISC-mediated processes in fibroblast cells. No substantial differences in specific mRNA copy number or localization were observed in the healthy cell line in comparison to mutant cell line (Figs. 1e, f, S1D). Therefore, we conclude that there are no significant differences in number or localization of normal vs. mutant variants of specific mRNA in human fibroblasts, although we were able to use only non-allele-specific quantification of transcripts by ddPCR and smFISH.

### The presence of the targeted region in an ORF and an *HTT*-specific flanking sequences improve the allele-selectivity of art-miRNAs

The results in human fibroblasts demonstrated that activity of art-miRNAs is dependent on specific features of targeted transcripts (Fig. 1a). PolyQ disease-related transcripts differ considerably in the lengths of their ORFs and UTRs as well as the localization of CAG repeat tracts. In details, CAG repeat tract is located in *HTT* and *ATXN7* at the 5′ end of the ORF, while in *ATXN3* at the 3′ end of the ORF, and in *ATN1* in the middle of the ORF (Fig. 2a). Therefore, we decided to elucidate how CAG repeat tract localization and sequences flanking CAG repeats influence silencing efficiency and allele preference by art-miRNAs. For this purpose, we generated pmirGLO-based plasmids encoding the *Fluc* gene, fused to a normal (~ 17 CAG repeats) or mutant (~ 65 CAG repeats) tract, and *Rluc* as internal reference (Fig. 2b). The repeat tract was placed at two sides of the *Fluc* ORF: at the *HTT*-like 5′ side (“5′ORF”) or at the *ATXN3*-like 3′ side (“3′ORF”), as well as in the 3′UTR, which is a typical region for miRNA-binding sites. Inserts contained either pure CAG repeats (P CAG) or ~ 50 nt-long *HTT* or *ATXN3* mRNA sequences flanking both sites of the CAG repeat tract, giving a total of 17 constructs (Fig. 2b).

First, we confirmed the expression of fusion proteins in HEK 293T cells (Fig. S2A). Next, we co-transfected the designed plasmids with selected oligonucleotides (art-miRNAs, siRNA targeting *Fluc*-siFluc or non-targeting siRNA-NTC) and performed a dual-luciferase assay. Typical siRNA, siFluc, caused efficient reduction of expression of all the constructs, to ~ 10% of the control level, regardless the location of the target site (Fig. S2C). In contrast, for A2 we observed varied activity for particular constructs (Fig. 2c). The most prominent downregulation of *Fluc* by A2 was obtained for the targeted sequence location in the “3′UTR” constructs (Fig. 2c, bars with blue background). The luciferase signal was decreased to ~ 35% of the control level regardless of repeat tract length and the sequence flanking CAG repeats. Downregulation of constructs expression with the target sequence localized in the ORF of *Fluc* was less efficient (Fig. 2c, bars with a red and green background), however for constructs containing *HTT* flanking sequence (both “3′ORF” and “5′ORF”) we observed significant allele-selectivity of A2 activity. In these cases, expression of the mutant construct was decreased to ~ 50% of the control level, whereas normal construct expression remained unchanged or decreased to only ~ 90% of the control level (Fig. 2c). Similar results were obtained for the other art-miRNAs analyzed: A4, G2 and G4 (Fig. S2b, c). Together, these observations demonstrate that the allele-selectivity of art-miRNAs was achieved only for constructs with *HTT*-specific sequences. In agreement with the results obtained in patient-derived fibroblasts (Fig. 1a), we conclude that better art-miRNAs allele-selectivity of *HTT* downregulation in comparison to *ATXN3*, is a combination of two effects: (1) increased downregulation of the mutant *HTT* allele as compared to mutant *ATXN3* and (2) decreased silencing of the normal *HTT* allele in comparison with normal *ATXN3*.

We also considered additional features of *HTT* and *ATXN3* mRNAs that could affect the discrepancy in art-miRNA allele-selectivity in HD and SCA3 models (Supplementary Text). For example, the presence of rare codons, upstream to miRNA-binding site, was shown to improve the efficiency of miRNA silencing for targets present in ORFs, possibly due to the decreased rate of translation [49]. Therefore, we analyzed the codon usage values in *HTT* and *ATXN3* transcript sequences upstream of the CAG repeat tracts (Fig. S3), but no significant differences were found for these mRNAs in this aspect (Supplementary Text).

### A2 and siHTT caused a decrease in *HTT* mRNA in the cytoplasm of HD NPs

**Fig. 3:**
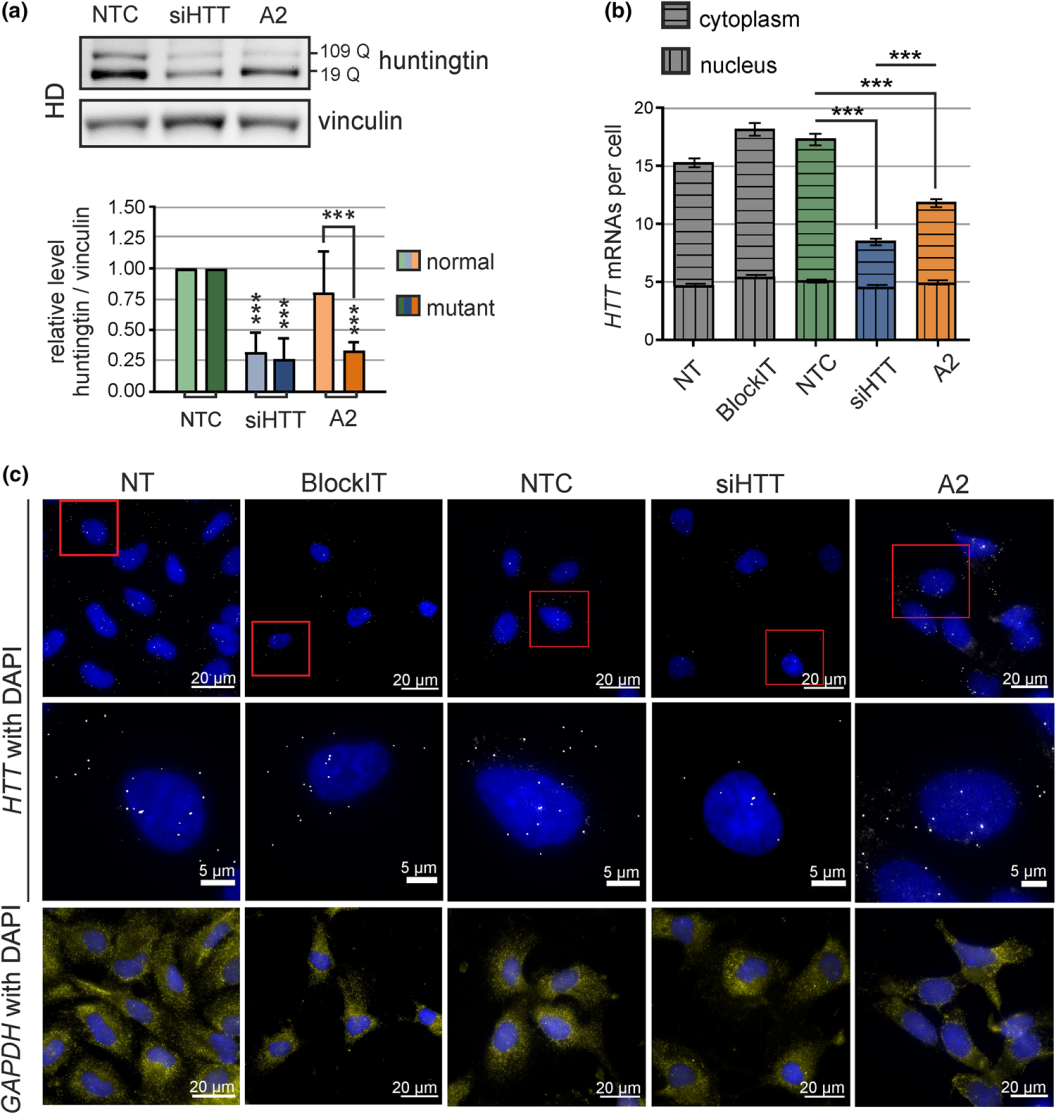
A2 activity in human HD neural precursors. **a** Western blot analysis of huntingtin allele levels in HD NPs lysed 48 h after transfection with 100 nM non-targeting siRNA (NTC, BlockIT siRNA), siHTT (siRNA for *HTT*) or the art-miRNA A2. *n* = 4. **b** Representative images showing the smFISH-based detection of *HTT* and *GAPDH* mRNAs in non-treated (NT) HD NP cells and HD NP cells treated with 100 nM fluorescent siRNA (BlockIT), non-targeting siRNA (NTC, siRluc), siHTT or A2. Cells were fixed 48 h after transfection with the indicated oligonucleotides. **c** Quantification of smFISH images for the experiments described in **b**. Signals were counted from at least 200 cells for each treatment. Data were analyzed using one-way ANOVA (with Bonferroni multiple comparisons test)

To investigate A2 activity in a more disease-relevant cell type, we included an additional model of human NP cells, derived from iPSCs. As A2 acted with high allele-selectivity for *HTT* silencing, we generated HD NPs. First, we characterized this cell line for the expression of neural stem cells markers (Fig. S4a, b) and optimized oligonucleotide delivery (Fig. S4c). Next, we investigated the efficiency of *HTT* expression silencing and changes in transcript abundance in HD NPs after transfection with *HTT*-specific siRNA (siHTT) or A2 art-miRNA. Similarly to results in HD fibroblasts (Fig. 1a), in HD NPs A2 allele-selectively downregulated mutant proteins to ~ 30% of the control level, without reduction in normal protein level, whereas siHTT decreased both alleles of the huntingtin protein to ~ 25% of the control level (Fig. 3a). Next, we performed a microscopic observation of endogenous *HTT* transcripts targeted with A2. Using non-allele-selective smFISH, we observed a cytoplasm-specific decrease in the huntingtin transcript number by ~ 45% after treatment with A2 and a more substantial reduction by ~ 70% after treatment with siHTT (Fig. 3b, c). This observation stays in agreement with the classical model of RISC activity in cytoplasm. Cellular localization of targeted transcript could affect the efficiency of its targeting by miRNA or siRNAs. In recent study, a larger fraction of *HTT* transcripts was detected in the nuclei of healthy human neuronal cells compared to non-neuronal cells [50]. In agreement with this observation, we also observed an increased ratio of nuclear to cytoplasmic *HTT* mRNAs in NPs, relative to fibroblasts (Figs. 1e, 3c). It is worth to notice that, after treatment with A2, we did not observe the retention of transcripts in the nucleus or cytoplasmic aggregation of mRNAs, that could result in decreased huntingtin synthesis and suggesting additional mechanisms of art-miRNA activity. Taken together, the results obtained in HD NPs show the therapeutic potential of A2, as its allele-selective activity was achieved not only in patient-derived fibroblasts but also in the neuronal cell line.

### Kinetic analysis of transcript and protein downregulation shows the early events of translation inhibition induced by A2

**Fig. 4:**
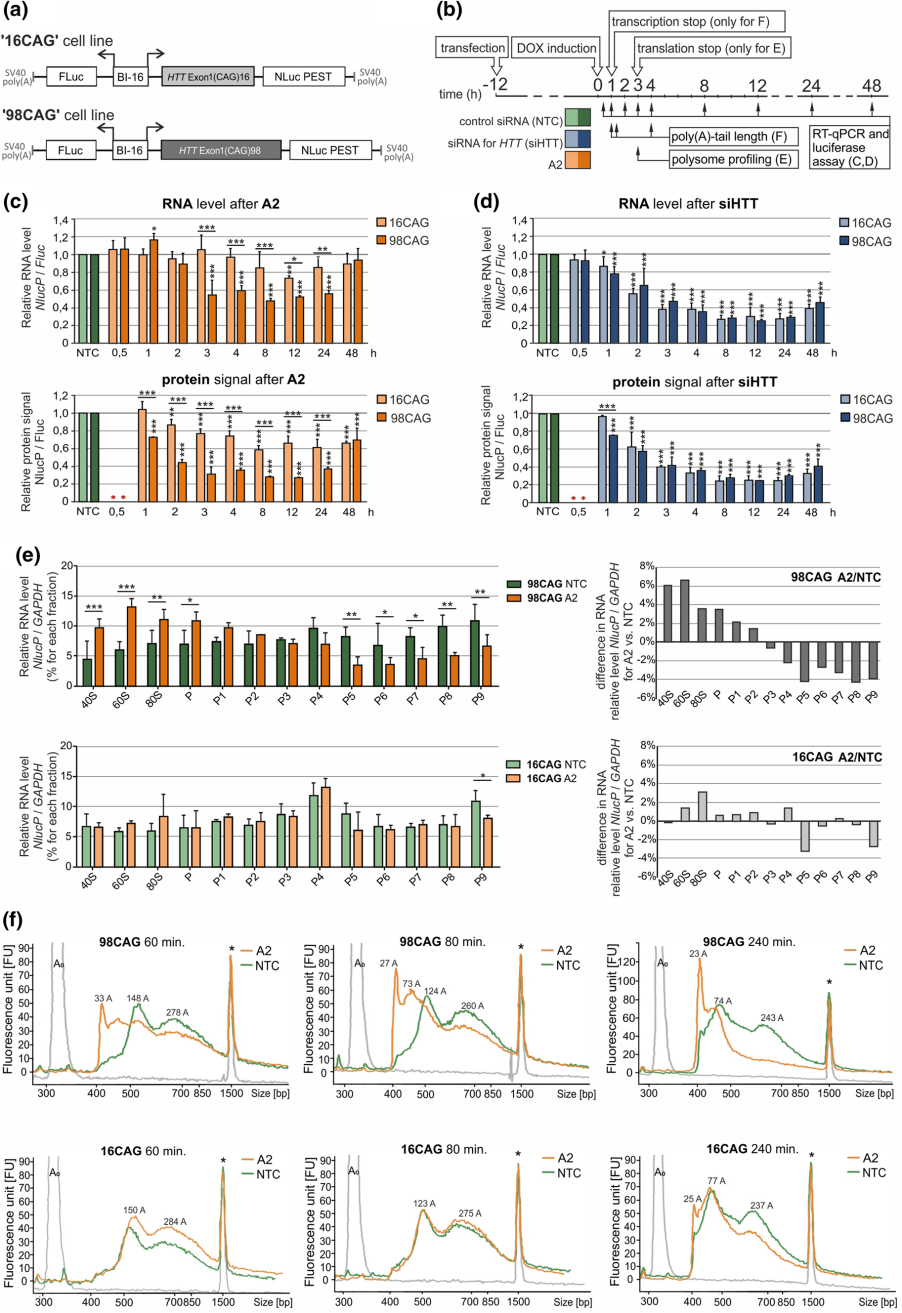
Mechanistic details of A2 activity in stable cell lines with inducible expression of the *HTT* fragment. **a** Constructs used to generate Flp-In T-REx-293 cell lines with two-directional, inducible expression of the *HTT* fragment (exon 1 with 16 or 98 CAG repeats) fused with *NlucP*. *Fluc* expression was used as a reference. **b** Timeline of the experiments presented in this figure. Specific treatment and cell lysis time points are indicated. **c**, **d** Results of RT-qPCR (upper panels) and dual-luciferase assay (lower panels) to determine the *HTT-NlucP* transcript level and HTT-NlucP protein signal, respectively, after transfection of the 16CAG and 98CAG cell lines with 100 nM A2 (**c**) or siHTT (**d**) at the indicated time points. The results were normalized to the mRNA level/protein signal of Fluc in the same sample and are shown as the relative expression level/relative protein signal of HTT-NlucP in cells transfected with 100 nM control siRNA (NTC, siRLuc). *n* = 3. **e** Results of RT-qPCR to assess *HTT-NlucP* expression levels in the indicated fractions containing ribosomal subunits (40S and 60S), the 80S monosome and polysomes (P-P9) after transfection of the 98CAG (upper panels) or 16 CAG (lower panels) cell lines with 100 nM A2 or control siRNA (NTC, siRLuc) at 3 h after induction. Data from each fraction were normalized to *GAPDH* expression and are presented as the % of *HTT-NlucP* expression in which 100% is the sum of the obtained values for all fractions*.* Graphs in the right panels show data with values calculated as the % difference in values obtained for separated fractions for control siRNA vs. A2. *n* = 3. The data for **c**–**e** were analyzed using two-way ANOVA. **f** Analysis of the poly(A) tail length of the *HTT-NlucP* transcript in the 98CAG (upper panels) or 16CAG (lower panels) cell lines at the indicated time points (60, 80, 120 min) after transfection with 100 nM A2 or control siRNA (NTC, siRLuc). Estimated poly(A) tail lengths are indicated. The experiment was repeated (*n* = 2), and similar results were obtained. *A*_0_—peak obtained with reporter-specific primers to amplify a region upstream of the polyadenylation site. *An internal standard peak (1500 bp upper marker)

To verify crucial factors affecting art-miRNAs activity and mechanistic details, we decided to include additional models with the exogenous expression of the targeted transcripts. For this purpose, we designed a dual-luciferase system for the inducible expression of *HTT* reporters. We generated Flp-In T-REx-293 cell lines stably expressing exon 1 of *HTT* (with 16 or 98 CAG repeats, hereafter called “16CAG” and “98CAG” cell lines, respectively) fused with the *NlucP* reporter, named *HTT-NlucP* (Fig. 4a). *Fluc* expression was used as a normalization control, and both reporters were placed under a bidirectional doxycycline-inducible promoter. First, we confirmed the similar expression of the reporters and non-significant Bl-16 promoter leakage in the absence of doxycycline (Fig. S5A, B). Next, we transfected the 16CAG and 98CAG cell lines with the art-miRNA A2, siRNA specific for *HTT*, siHTT, or non-targeting siRNA, followed by induction of reporter expression and subsequent analysis of the transcript and protein levels at particular time points (Fig. 4b).

The kinetics of *HTT* reporter transcript and protein downregulation by A2 were clearly different in the 16CAG and 98CAG cell lines (Fig. 4c). For the mutant *HTT* reporter transcript, we observed a maximum of ~ 50% downregulation starting 3 h after induction, whereas for the normal *HTT* reporter transcript, we detected only a slight decrease (Fig. 4c, upper panel). In contrast to the transcript levels, repression of the HTT-NlucP protein by A2 was more prominent, and the mutant protein level was decreased up to ~ 30%, while the normal protein was decreased up to ~ 60% of the control level at selected time points (Fig. 4c, lower panel). Interestingly, at early time points (up to 2 h post-induction), A2 significantly lowered only the level of mutant protein, suggesting that translational repression preceded mRNA decay in the allele-selective inhibition of the mutant *HTT* allele. As a reference for the typical RNAi mechanism, we performed the same analysis with siHTT. We observed rapid transcript and protein downregulation with no apparent difference in activity towards the normal and mutant alleles (Fig. 4d), suggesting the AGO2-mediated cleavage of both transcripts and, as a result, a decrease in the protein levels.

### A2 causes a shift of mutant transcripts from heavy polysome fractions

To assess translation inhibition caused by art-miRNA in more detail, we performed polysome profiling analysis of *HTT* reporter transcripts. The 98CAG and 16CAG cell lines were treated with A2 or control siRNA, and lysates were prepared 3 h after the induction of luciferase expression (Fig. 4b, e, representative UV absorbance profiles, including a profile following disruption with EDTA, are shown in Fig. S6a). In 98CAG cells A2 caused a statistically significant shift in *HTT* reporter transcript distribution in the analyzed fractions, as referred to non-targeting siRNA treatment. We observed an approximately two-fold change in mutant *HTT-NlucP* transcript abundance in selected fractions, i.e., increased cosedimentation with 40S, 60S, 80S and first light polysome fraction and decreased cosedimentation with heavier polysomes, as compared to the treatment with control siRNA (Fig. 4e, upper panels). This results suggest that A2 inhibited mutant *HTT-NlucP* translation at initiation and/or early elongation step. In these experiments, some *HTT-NlucP* transcripts remained associated with heavier polysomes after A2 treatment, probably because not all *HTT* reporter transcripts were bound by this art-miRNA. Our conclusions are supported by analogous control experiments performed in the 16CAG cell line which results did not show any significant difference in the cosedimentation of *HTT-NlucP* transcripts across the collected fractions between A2- and control siRNA-treated cells (Fig. 4e, lower panels). Moreover, as expected, no significant changes in *Fluc* expression relative to *GAPDH* expression were observed in experiments using both, 98CAG and 16CAG cell lines (Fig. S6b). Together, we conclude that observed translation inhibition occurred very rapidly and efficiently for mutant transcript as a result of A2 activity.

### A2 induces rapid shortening of the targeted mRNA poly(A) tail

We aimed to explain in more detail the observation that mutant *HTT* reporter protein level was decreased after A2 treatment already at early time points (1–2 h) after induction, without a change in the level of its transcript (Fig. 4c). This can be explained as the effect of direct translation inhibition or transcript deadenylation, which in turn results in reduced translation due to disrupted transcript circulation. To verify the latter mechanism, we performed transcription pulse-chase experiment and examined the length of poly(A) tails in *HTT* reporter transcripts using poly G/I extension followed by resolution of the PCR products in a microfluidic chip (Fig. S7a). In details, after transfection of the 98CAG and 16CAG cell lines with A2 or control siRNA, we induced expression of the *HTT* reporter for 1 h, stopped the transcription and then analyzed poly(A) tail length profiles at three time points (Fig. 4b, f).

In the 98CAG cell line, A2 caused significant deadenylation of *HTT-NlucP* transcript already at the 60 min time point, when a substantial pool of *HTT* reporter transcript deadenylation intermediates with a short (~ 30 A) poly(A) tail appeared (Fig. 4f, upper panels). The formation of transcripts with shortened poly(A)-tails further accelerated at the 80 min and 240 min time points (Fig. 4f, upper panels). In contrast, we did not observe these deadenylation intermediates at even the 240 min time point after treatment of the 98CAG cell line with control siRNA. Analogous experiments in the 16CAG cell line showed no significant difference in the poly(A) tail length profiles of A2- and control siRNA-treated cells at the 60 and 80 min time points and only a slight difference at the latest 240 min time point (Fig. 4f, lower panels). In both cell lines and following treatment with A2 and control siRNA, we observed significant changes in the length of the poly(A) tail at 240 min compared to that at earlier time points (Fig. S7b), indicating events typical of transcript decay after transcription arrest. Taken together, A2 caused rapid deadenylation only of mutant *HTT-NlucP* transcript, suggesting crucial role of this process in the art-miRNA-mediated repression. Deadenylation is expected to lead to mutant transcript degradation observed at later time points (3–24 h) (Fig.4c).

### AGO2 is dispensable for the A2-mediated silencing of *HTT* expression

**Fig. 5:**
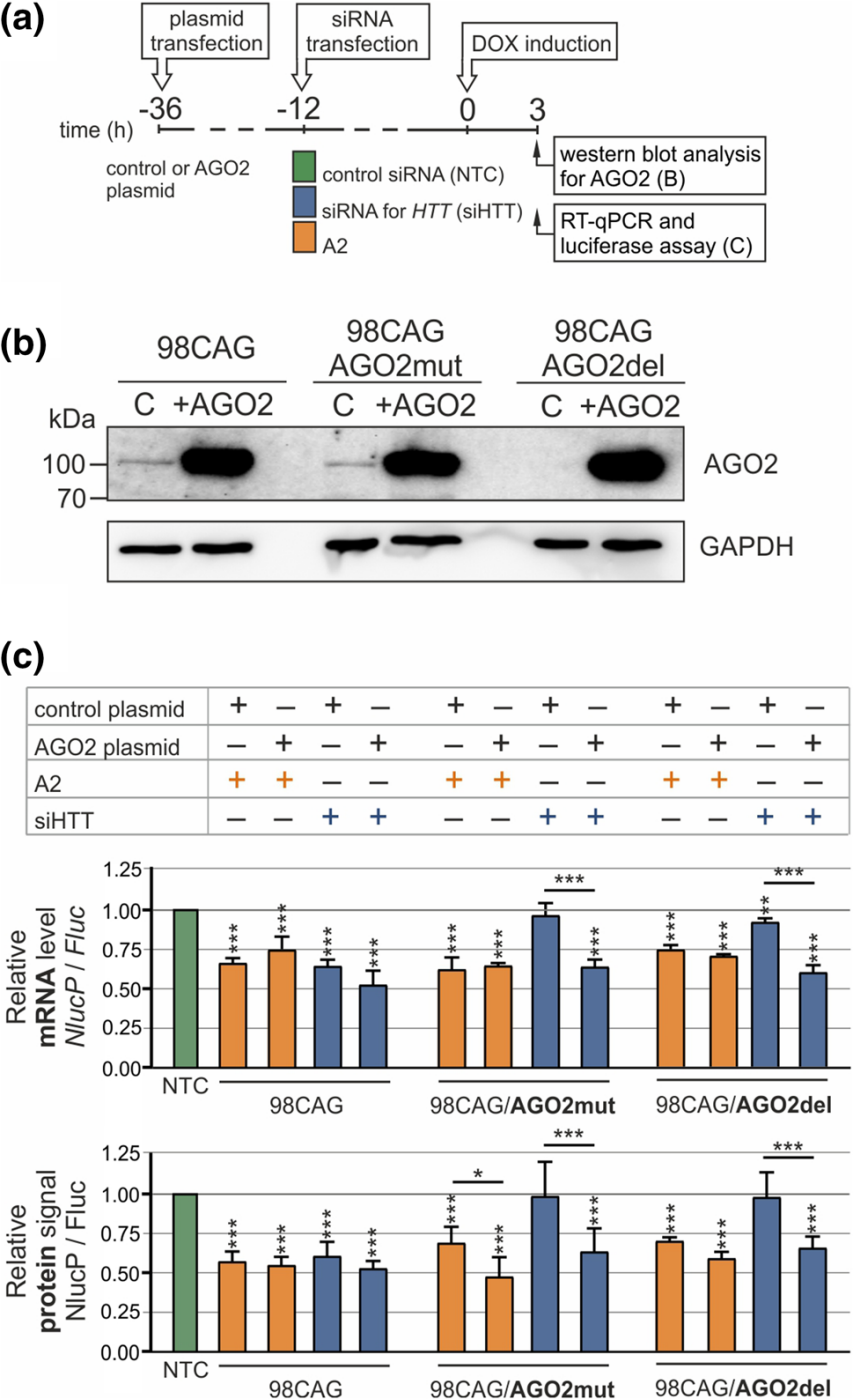
Verification of the involvement of AGO2 in A2 activity. **a** Timeline for the experiments presented in this figure. Specific treatment and cell lysis time points are indicated. **b** Western blot analysis of AGO2 protein levels in 98CAG Flp-In T-REx-293 stable cell lines (*98CAG* standard cell line, *AGO2del* deletion of endogenous AGO2, *AGO2mut* abolished catalytic activity of slicer domain). *+AGO2* cell lines after transfection with AGO2 WT plasmid to rescue protein. **c** Results of RT-qPCR and luciferase assays to detect *HTT-NlucP* mRNA and protein levels, respectively, after transfection of the 98CAG cell lines (including AGO2mut and AGO2del) with 100 nM A2 or siHTT as well as the indicated plasmids. Data were normalized to *Fluc* expression levels in the same sample and *HTT-NlucP* expression levels after transfection with 100 nM control siRNA (NTC, siRLuc). The data were analyzed using two-way ANOVA (with Bonferroni multiple comparisons test among a set of samples for each of the cell lines). *n* = 3

Our next step was to elucidate the role of AGO2 in art-miRNA mechanism. We wanted to determine if AGO2 presence or the activity of its catalytic subunit is required for art-miRNAs-mediated silencing. To verify this, we modified the 98CAG cell line using CRISPR-Cas9 technology. We created homozygous cell lines in which *AGO2* gene was knocked out (AGO2del) or a cleavage-deficient AGO2/D597A protein was expressed (AGO2mut) (Fig. S8a, b). The D597A mutation in AGO2 is known to abolish RNA cleavage without affecting efficiency of siRNA binding or translational repression [51, 52]. Two sgRNAs were designed to knock out AGO2 by deletion of a gene fragment in exon 2 leading to premature STOP codons (Fig. S8a, b), whereas an approach using one sgRNA and a donor template containing a specific nucleotide mutation was used to introduce a catalytic mutation in AGO2 (Fig. S8c, d). We selected the final clones based on the results of DNA sequencing (Fig. S8b, d) and AGO2 immunoblotting (Fig. 5b).

To analyze the requirement of AGO2 in A2 and siHTT activities, we analyzed both the transcript and protein levels of the mutant *HTT* reporter in 98CAG-AGO2mut and -AGO2del cell lines. In addition, we performed rescue experiments and transfected cell lines with plasmids encoding WT AGO2 (Fig. 5b). Based on previous experiments (Fig. 4c), we selected early time point of 3 h after the induction of *HTT-NlucP* expression (Fig. 5a) which we found suitable for analysis of the details of A2 activity. As in previous experiments, in this time point in the 98CAG cell line (Fig. 4c, d), both A2 and siHTT repressed *HTT-NlucP* expression up to 50% of control level (Fig. 5c). As expected for the AGO2mut and AGO2del cell lines transfected with typical siRNA, siHTT, repression of *HTT-NlucP* was completely abolished at both the transcript and protein levels and could be restored after WT AGO2 overexpression (Fig. 5c, blue bars). In contrast, after A2 transfection into AGO2mut and AGO2del cell lines, efficient lowering of both, transcript and protein levels of HTT-NlucP was achieved (Fig. 5c, orange bars). Additionally, we observed no substantial effects of WT AGO2 overexpression on A2 and siHTT activities. Together, our results show that A2-mediated downregulation of *HTT* reporter expression is mostly independent of AGO2-mediated slicer activity but is rather a consequence of transcript deadenylation and translation inhibition. Moreover, considering canonical miRNA-related mechanisms, our observations suggest that other AGO proteins (AGO1, AGO3, AGO4) and their respective miRISCs are sufficient in mutant transcript repression caused by A2, in the absence of AGO2.

## Discussion

### Normal and mutant alleles of polyQ diseases-related genes show varied susceptibility to regulation by art-miRNAs

Art-miRNAs were designed to target mutation site, i.e. expanded CAG repeat tract, in several transcripts implicated in polyQ diseases [25, 26]. The main rationale behind such design was to generate the universal treatment for these rare disorders. However, during research we and others (Supplementary Table 1) observed varied susceptibility of targeted transcripts to regulation by art-miRNA (Fig. 1a).

The common feature of polyQ diseases-related mRNAs is their rather low cellular level, including the brain regions mostly affected in polyQ diseases [53]. We confirmed the relatively low expression of four selected genes in patient-derived fibroblasts (*HTT*, *ATN1*, *ATXN3* and *ATXN7*), however, we also found substantial differences between the quantities of these mRNAs (Fig. 1d). In previously published studies, turnover rate and cellular abundance of transcripts were found to influence the efficiency of downregulation mediated by RNAi [54, 55]. Therefore, these factors might contribute to the observed differences in A2 activity for various transcripts, i.e., higher efficiency of *ATN1* silencing may result, at least partially, from higher expression level of this gene.

In addition to the cellular factors, we looked at polyQ disease-related transcripts and noticed that they differ in their arrangement of specific regions as well as the location of the repeat tract within the ORF (Fig. 2a). To address specific questions concerning the impact of CAG repeat tract localization on differences in allele-selective silencing by art-miRNAs, we developed cellular models with exogenous expression of the designed constructs (Fig. 2b). Silencing of exogenes expression may differ from that of endogenes, as higher expression levels of exogenes are obtained, and all additional sequences that could affect silencing efficiency are only present in endogenes. Nevertheless, we observed clear tendencies in the potency of art-miRNAs depending on the location of their targeted site and its flanking sequences (Fig. 2c). Clearly, CAG repeat tract length also determines art-miRNA activity, as large differences were observed for normal and mutant alleles silencing. Moreover, we revealed that it is crucial that the targeted sequence is present in the ORF, as the presence of the targeted tract in the 3′UTR caused efficient silencing of both normal and mutant alleles (Fig. 2c). Possibly this preference is caused by the lack of ongoing translation in 3′UTRs, in contrast to ORF regions where ribosomes interfere with the miRISC complexes [56]. Additionally, unique features of each transcript and the context of the targeted sequence can affect efficiency and allele-selectivity of art-miRNAs. One such factor may be the structure formed by the CAG repeat tract, the stability of which was shown *in vitro* to be dependent on the flanking sequence (reviewed in [57]). Moreover, additional factors, like varying distance of the targeted site from STOP codon or from 3′ and 5′-ends of transcript (Fig. 2a), could contribute to diversity in efficiency of polyQ diseases-related genes silencing by A2 [58, 59].

### Art-miRNAs activate events of mRNA deadenylation and translation inhibition

MiRNA-mediated regulation is known to occur in many ways depending on the activating miRNA and targeted mRNA, which can affect each other through multiple miRISC components [60, 61]. As the repertoire of miRISC proteins activate various cellular processes, the detailed analysis of a particular gene silencing mechanism is complex [3, 62]. Briefly, this silencing mechanism involves AGO-mediated recruitment of the GW182/TNRC6 protein family [51, 63], followed by subsequent binding of poly(A)-binding protein (PABPC), mRNA deadenylase complexes PAN2-PAN3 and CCR4-NOT, catalyzing deadenylation of the mRNA target, eventually leading to target decapping and transcript degradation ([64–67], reviewed in [2]). We show that art-miRNAs targeting expanded CAG repeats in ORF regions cause translation inhibition (Fig. 4e, top panel) and activate rapid mRNA deadenylation (Fig. 4f, top panel), similarly to classical miRNA pathway. Moreover, mRNA deadenylation and translational repression in typical miRISC-mediated gene silencing were also shown to be interconnected when AGO-miRNAs bound 3′UTRs (reviewed in [3, 62]). Nevertheless, the vast majority of endogenous miRNAs target 3′UTR sequences and cause mRNA decay (estimated at 66–90%) that is directly responsible for the protein downregulation [62, 68, 69]. In this study, we initially observed stronger lowering of protein level, than mRNA level (Fig. 4c), suggesting that for a pool of transcripts targeted by art-miRNAs in ORF region, translation was inhibited without activation of mRNA decay. However, when we performed detailed poly(A) tail length analysis after A2 art-miRNA treatment, deadenylation was observed already at the earliest time point analyzed after transgene induction (60 min) (Fig. 4f, top panel). On the other hand, our data obtained from polysome profiling suggested that A2-activated translational repression occurs also very rapidly, 3 h after transgene induction (Fig. 4e, top panel). Therefore, we cannot exclude that deadenylation preceded or was concurrent with translation inhibition. Indeed, in some cases, translational inhibition was shown to precede poly(A) tail shortening and mRNA decay. Such cases were: a study performed in HeLa cells [8] and a study using a Drosophila S2 cell-based controllable expression system [70] where miRNA-targeted sequences were localized in the 3′UTR. Here, to understand the investigated mechanism, it was also crucial to determine if ribosome complexes are formed on targeted transcripts as the result of art-miRNA activity. Our data obtained from polysome profiling suggest that A2-activated translational repression can occur at the elongation step and/or at the initiation step (Fig. 4e). This conclusion is supported by the comparison of the shift of *HTT* transcript, observed after A2 treatment, with the profiles of mRNA distributions in monosome and polysome fractions characteristic for global inhibition of translation at the initiation or elongation step [71, 72].

**Fig. 6:**
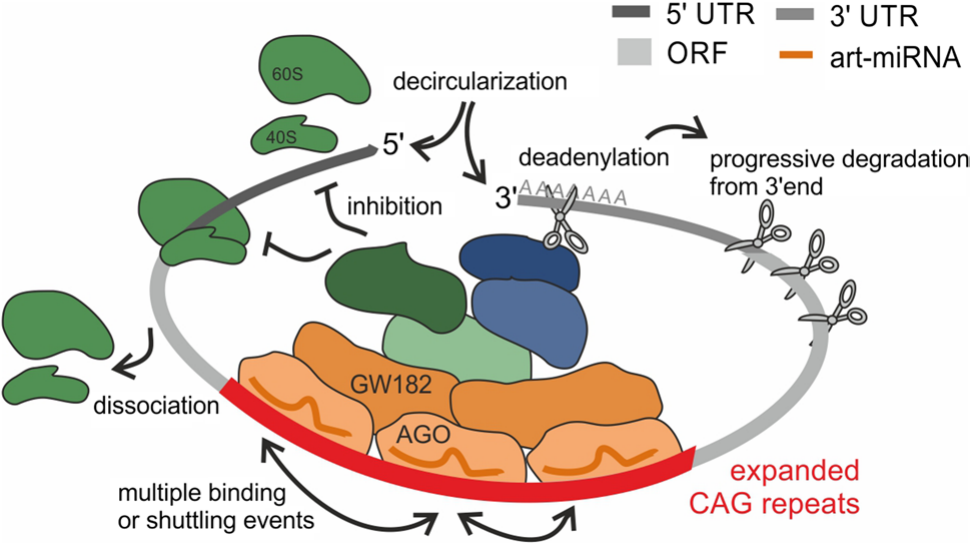
Model of art-miRNA activity targeting transcripts containing expanded CAG repeat tracts within the ORF region. Art-miRNA loaded into AGO binds to mutant CAG repeat tract with a mismatch formed in the central region of this interaction. Multiple binding or shuttling of the AGO protein results in the formation of the miRISC, which affects translation by the inhibition of its initiation or early elongation. Shortening of the poly(A) tail is activated for a pool of targeted transcripts that leads to the subsequent degradation of mRNA

Transcript-dependent factors, affecting the activation of deadenylation and translation inhibition processes, may also contribute to differences in the effectiveness of art-miRNA in different models of polyQ diseases. According to translation-dependent closed-loop model [73, 74], it can be assumed that, although CAG repeats are in a very large distance from the poly(A) tail (especially for *HTT* transcript ~ 13 kb), art-miRNA-bound miRISCs are in close proximity to the poly(A) tails of polyQ disease-related mRNAs in cells (Fig. 6). In this case, miRISC-mediated translational repression can also occur through recruitment of the RNA helicase DDX6, which acts as both a translational inhibitor and decapping activator [75–77]. Moreover, it was also shown that DDX6 can act by displacing the eukaryotic translation initiation factors eIF4A-I and eIF4A-II from the targeted transcripts, thereby preventing translation initiation [10, 78, 79]. These processes are dependent on features of 3′UTR region of targeted transcript, as various cis- and trans-acting elements in specific 3′UTRs were found to influence miRNA-mediated gene expression regulation [80].

### The art-miRNA mechanism as a model for the AGO-dependent cooperative activities of miRNAs within ORF regions

It is known that miRNAs regulate genes expression mostly by recognition of sites within 3′UTR, however numerous miRNA-binding sites were revealed by global approaches also in the ORFs of human mRNAs [81, 82]. The functionalities of approximately twenty sites of this type in specific transcripts have been experimentally confirmed so far (reviewed in [83]), but precise mechanisms have not been extensively investigated. Recently, a specific type of miRNA recognition elements exclusive to ORF regions was described, and a mechanism of gene expression regulation by temporary ribosome stalling was proposed for DAPK3 kinase [84]. Based on the results of our study, we can extrapolate mechanistic details of ORF regions-targeting by miRNAs, especially when multiple binding sites are present. Indeed, for miRNA-based regulation within ORFs, multiple binding sites have been found frequently [83]. One such example is the regulation of the expression of a family of genes containing C_2_H_2_ zinc-finger domains by a group of miRNAs [85, 86]. Additionally, a general role of repeat tracts localized in ORFs in post-transcriptional regulation was suggested based on predicted interactions [86]. Overall, these data and results regarding art-miRNA activity suggest that for the efficient regulation of a gene’s expression by targeting its ORF, multiple binding sites are required, resulting in cooperative action. Some mechanistic details of the cooperative activity of miRNAs were revealed, e.g., a FRET-based method was used to show that AGO2 dissociation is in kinetic competition with lateral diffusion, resulting in shuttling between adjacent target sites [87].

In general, human cells express four AGO paralogs (AGO1-4) that act to regulate miRNA-based gene expression [88]. AGO2 is the most abundant, as it accounts for ~ 70% of the total AGO pool in HEK 293T and fibroblast cells [89, 90], in which we performed most of mechanistic experiments. Previously, discrimination between the normal and mutant alleles of *HTT* mRNA by art-miRNAs was reported to be highly sensitive to the cellular pool of AGO2 and GW182 family proteins [30]. Our analysis in total AGO2 knockout and AGO2 D597A endonuclease-deficient cell lines, showed that the absence of AGO2 does not affect the observed silencing activity of exemplary A2 art-miRNA (Fig. 5c). These results suggest that other slicer-deficient AGOs (AGO1, 3 and/or 4) can act in the cooperative repression of the mutant allele. Nevertheless, we do not rule out that AGO2 is a key, most abundant miRISC core protein, but we show it may be replaced by other AGOs. These results are consistent with previous observations that the majority of human miRNAs associate with all four AGOs and do not have a preference for a particular AGO paralog [20, 91, 92]. We assume, that after art-miRNA-AGO complex binding to mRNA, the subsequent co-recruitment of the GW182/TNRC6 with other effector miRISC proteins results in allele-selective inhibition of the mutant allele. Our data suggest that art-miRNAs can be additionally recruited by slicer-deficient AGO proteins (AGO1, 3 and/or 4) to the expanded CAG repeats (Fig. 6) what might turn out to be advantageous for the experimental therapy based on art-miRNA reagents. This is due to the previous RNA-seq and mass spectrometry analysis that clearly indicate that in brain tissue the relative abundance of AGO1, AGO3 and/or AGO4 (when related to the total AGO pool) are higher than in many other cells and tissues analyzed [89, 93]. This particularly applies to AGO1 protein as quantitative proteomic approach revealed in HEK 293T cells the following proportions of AGO proteins: ~ 17% AGO1, ~ 75% AGO2, ~ 6% AGO3 and ~ 2% AGO4, whereas similar mass spectrometry analysis conducted on mouse brain lysates showed proportions: ~ 35% AGO1, ~ 55% AGO2, ~ 9% AGO3 and < 1% AGO4 [89].

### Art-miRNA activity in the context of other therapeutic strategies for polyQ diseases

We are now witnessing large advances in antisense oligonucleotide (ASO)- and RNAi-based strategies in clinical trials [94, 95]. The most advanced clinical trial of a causative therapy for HD involves the intrathecal delivery of RNase-H-activating ASOs targeting *HTT* (ClinicalTrials.gov Identifier: NCT03842969). The results obtained thus far are very promising, as a decrease in huntingtin in the spinocerebellar fluid was reported [96]. Nevertheless, many challenges remain to be faced in the developed therapy, among which the allele-selectivity of silencing is one of the major points [97]. Preservation of the level of the normal allele might be required, as its long-term downregulation, which would be the result of long-term treatment, could have many adverse effects [98, 99]. Additionally, in the case of HD, some reports suggest that targeting exon 1 of *HTT*, which contains the CAG tract, may be crucial to eliminate key toxic entities causing HD pathogenesis [100, 101]. For these reasons, we find an approach using art-miRNAs to be desirable. This CAG repeat-targeting strategy offers an option for the preferential silencing of several mutant alleles responsible for polyQ diseases and would be applicable to a larger group of patients than an allele-selective SNP-targeting-based approach. Moreover, due to somatic instability, mosaicism of highly expanded CAG repeats in the brain is likely a common effect largely responsible for brain-specific pathology, as shown in HD [102–104]. In this case, transcripts containing increasingly expanded CAG repeat tracts are expected to be more efficiently targeted by art-miRNAs. This assumption remains to be proven experimentally but would clearly allow preferential targeting of the RNAs which translation leads to pathogenesis.

The potential universality of targeting the mutation site in RNA was also shown for the activity of ASOs acting as translation blockers or splicing modulators that were tested for several polyQ diseases [105–107]. According to recent findings, an art-miRNA-based therapeutic strategy might not be applicable for SCA1 [108], and the development of a universal molecule for several polyQ diseases might require further research. Although more demanding than initially assumed, the applicability of this strategy in at least a few disorders remains feasible. Art-miRNAs possess additional advantages as they can be chemically modified [31, 38] or expressed from vectors [33, 109], and can include application of novel approaches for delivery to the brain [110, 111]. Interestingly, CRISPR-Cas9- and ZFP-based CAG repeat-targeting strategies were recently successfully tested in HD models [112–116]. These approaches offer an alternative solution for mutant *HTT* inhibition after the binding of specifically designed molecules to mutant DNA, but some challenges remain before their clinical testing.

In summary, our model of art-miRNAs activity show potential versatility in the miRNA-based regulation of gene expression. Although this model (Fig. 6) is based on results obtained for artificial miRNA, it contributes to a better understanding of the mechanisms of action of natural miRNAs which interact with sequences located in ORFs with adjacent multiple binding sites. These mechanisms have been harnessed to activate the therapeutically beneficial silencing of mutant genes with CAG repeat expansions, showing the great flexibility of RNAi-based mechanisms in cells.

### Electronic supplementary material

Below is the link to the electronic supplementary material.

Supplementary file1 (DOCX 3704 kb)

## Abbreviations

16CAG cell line: Stably expressing exon 1 of *HTT* with 16 CAG repeats
98CAG cell line: Stably expressing exon 1 of *HTT* with 98 CAG repeats
art-miRNAs: CAG repeat-targeting artificial miRNAs
ASO: Antisense oligonucleotide
*ATN1*: Atrophin-1
*ATXN3*: Ataxin-3
*ATXN7*: Ataxin-7
ddPCR: Digital droplet PCR
DRPLA: Dentatorubral–pallidoluysian atrophy
Fluc: Firefly luciferase
*HTT*: Huntingtin
HD: Huntington’s disease
ICC: Immunocytochemistry
iPSCs: Induced pluripotent stem cells
miRNA: MicroRNA
NP: Neural progenitor
ORF: Open reading frame
polyQ: Polyglutamine
RISC: RNA-induced silencing complex
SCA3: Spinocerebellar ataxia type 3
sgRNA: Small guide RNA
siRNA: Short interfering RNA
smFISH: Single-molecule fluorescent in situ hybridization
Rluc: *Renilla* Luciferase
NlucP: Nano luciferase with PEST domain
RT-qPCR: Quantitative reverse transcription PCR
UTR: Untranslated region
