# Supplementary material for: Artificial miRNAs targeting CAG repeat expansion in ORFs cause rapid deadenylation and translation inhibition of mutant transcripts
Source: Cell Mol Life Sci. 2020 Jul 21;78(4):1577–96. doi: 10.1007/s00018-020-03596-7 (PMC7904544; doi:10.1007/s00018-020-03596-7)
Supplement: Supplementary file 1 — Supplementary file1 (DOCX 3704 kb) [file 18_2020_3596_MOESM1_ESM.docx]

**SUPPLEMENTARY DATA**

**SUPPLEMENTARY TEXT**

**Features of *HTT* and *ATXN3* transcripts which may affect allele-selective regulation by art-miRNAs**

The results of luciferase assay (Fig. 2C) suggest that within sequence of *HTT* transcript there are “positive factors” for allele-selective silencing (decreasing silencing of normal allele and/or increasing silencing of mutated allele), whereas within *ATXN3* mRNA some “negative factors” can be present. We analyzed *HTT* and *ATXN3* transcripts with the use of bioinformatic approach, i.e. RegRNA 2.0 web server for identifying functional RNA motifs and binding sites [1]. RegRNA for *ATXN3* transcript sequence around CAG repeat tract did not show any potential binding sites for molecules. For *HTT* mRNA we identified miR-3960 binding site with near fully complementary binding, including lack of mismatches in seed region and single mismatch in central region. Nevertheless, experimental validation showed that miRNA-3960 did not improve significantly silencing efficiency of art-miRNAs (data not shown).

Moreover, we checked the frequency of codons upstream of CAG repeat tract within tested transcripts (*HTT*, *ATXN3* and *Rluc*) (Fig. S3). The analysis is based on observation that translation inhibition efficiency by miRNAs targeting ORF regions may dependent on the presence of rare codons from the translation start to miRNA binding site [2]. Slowing down the ribosomes on the transcript, caused by the translation of rare codons, was shown to increase downregulation efficiency by miRNAs targeting ORF regions [2]. Three analyzed transcripts contained codons with average codon usage values similar to optimal codons used by Gu et al. However, for comparison of *HTT* and *ATXN3* transcripts we did not observed significant differences in their average codon usage values for regions upstream to CAG repeats. Therefore these analyses do not justify observed differences in allele-selectivity in art-miRNAs activity in HD and SCA3 models.

**SUPPLEMENTARY MATERIALS AND METHODS**

**Immunocytochemistry (ICC)**

NP cells were fixed in 4% PFA, permeabilized with 0.5% Tween and blocked in 1% bovine serum albumin (all in 1xPBS), followed by incubation with primary antibodies and fluorescent-dye conjugated secondary antibodies (after wash in PBS), listed in Supplementary Table 3. DAPI was used for nuclei staining. Images were captured with Leica DMI6000 microscope as described in Methods section.

**Details on generation of pcDNA5/FRT/TO-based constructs for inducible and stable expression of CAG repeat target sequence**

Each intermediate stage of cloning and the final modified pcDNA5/FRT/TO-based plasmids were confirmed by Sanger DNA sequencing. All PCR fragments used for cloning were obtained with Q5 High-Fidelity DNA Polymerase (New England BioLabs) according to manufacturer’s instructions. Sequences of DNA oligonucleotides used for cloning are given in Supplementary Table 7. The following steps were performed:

1) *Fluc* sequence was PCR amplified from pmirGLO plasmid (Promega) using primers (1/2). Next, the *Fluc* fragment was digested with KpnI/BamHI restriction enzymes, and cloned between KpnI/BamHI sites in pcDNA3.1(+) plasmid (Invitrogen) to obtain Fluc_pcDNA3.1(+) plasmid.

2) SV40pA was PCR amplified from pNL1.2 plasmid (Promega) using primers (3/4). Next, the SV40pA fragment was digested with BglII/BamHI restriction enzymes, and cloned, in a correct orientation, between BamHI site in Fluc_pcDNA3.1(+) plasmid to obtain Fluc_SV40pA pcDNA3.1(+) plasmid.

3) Fluc_SV40pA_pcDNA3.1(+) plasmid was digested with KpnI/BamHI restriction enzymes to obtain Fluc_SV40pA fragment. Next, the Fluc_SV40pA insert was cloned, in reverse orientation, between BglII/KpnI sites in pcDNA5/FRT/TO plasmid (Invitrogen) to obtain SV40pA_Fluc pcDNA5/FRT/TO plasmid.

4) The generation of bidirectional tetracycline-inducible BI-16 promoter, a derivate of the original CMV promoter duplicated back-to-back was done as in [3] with minor modifications. Asymmetric fragments 1 and 2 of PCMV(2x-TetO2) promoter from pcDNA5/FRT/TO were amplified by PCR and then ligated together. Fragment 1 was amplified using primers (5/6), while fragment 2 was amplified using primers (7/8). PCR products were cut with XbaI, mixed and ligated using T4 DNA ligase to make an asymmetric inverted repeat. The intermediated-length products of these ligations, representing the heterodimers were purified from agarose gel. Next, the bidirectional promoter fragment was digested with KpnI/BamHI and inserted between KpnI/BamHI digested SV40pA_Fluc pcDNA5/FRT/TO plasmid to generate SV40pA_Fluc_BI-16 pcDNA5/FRT/TO plasmid.

5) SV40pA was PCR amplified from pNL1.2 plasmid using primers (9/10). Next, the SV40pA fragment was digested with BclI/SphI restriction enzymes, and cloned between BclI/SphI sites in pcDNA5/FRT/TO plasmid to obtain SV40pA pcDNA5/FRT/TO plasmid.

6) *NlucP* sequence was PCR amplified from pNL1.2 plasmid using primers (11/12). Next, the *NlucP* fragment was digested with XhoI/ApaI restriction enzymes, and cloned between XhoI/ApaI sites in SV40pA_pcDNA5/FRT/TO plasmid to obtain NlucP_SV40pA pcDNA5/FRT/TO plasmid.

7) Oligo 13 and 14, as well as, oligo 15 and 16 were annealed to generate Linker 13/14 and ATGLinker 15/16, respectively. Linker 13/14 was inserted, in a correct orientation, between XhoI site in NlucP_SV40pA pcDNA5/FRT/TO plasmid to obtain Linker_NlucP_SV40pA pcDNA5/FRT/TO plasmid. ATGLinker 15/16 was inserted between BamHI/XhoI sites in NlucP_SV40pA pcDNA5/FRT/TO plasmid to obtain ATGLinker_NlucP_SV40pA pcDNA5/FRT/TO plasmid.

8) ATGLinker_NlucP_SV40pA pcDNA5/FRT/TO plasmid was digested with BamHI/SphI restriction enzymes to obtain ATGLinker_NlucP_SV40pA fragment. Next, the ATGLinker_NlucP_SV40pA insert was cloned between BamHI/SphI sites in SV40pA_Fluc_BI-16 pcDNA5/FRT/TO plasmid to obtain SV40pA_Fluc_BI-16_ATGLinker_NlucP_SV40pA pcDNA5/FRT/TO final plasmid.

9) Linker_NlucP_SV40pA pcDNA5/FRT/TO plasmid was digested with XhoI/SphI restriction enzymes to obtain Linker_NlucP_SV40pA fragment. Next, the Linker_NlucP_SV40pA insert was cloned between XhoI/SphI sites in SV40pA_Fluc_BI-16 pcDNA5/FRT/TO plasmid to obtain SV40pA_Fluc_BI-16_Linker_NlucP_SV40pA pcDNA5/FRT/TO plasmid.

10) Sequence of *HTT* exon 1 with 16 CAG or 98 CAG repeats were PCR amplified using (17/18) primers from previously obtained *HTT* cDNA clones (from Coriell Repositories cell line NM_002111). Next, both the amplicons were digested with BamHI/XhoI restriction enzymes, and cloned between BamHI/XhoI restriction sites in SV40pA_Fluc_BI-16_Linker_NlucP_SV40pA pcDNA5/FRT/TO plasmid to obtain SV40pA_Fluc_BI-16_HTT16_Linker_NlucP_SV40pA pcDNA5/FRT/TO and SV40pA_Fluc_BI-16_HTT98_Linker_NlucP_SV40pA pcDNA5/FRT/TO final plasmids respectively.

**Details of generation of Flp-In T-REx-293 cell lines**

Flp-In T-REx-293 Host Cell line was grown in 21 cm^2^ dishes to ~70% confluence. Next, the cells were co-transfected with a mixture containing pcDNA5/FRT/TO-based expression vector and pOG44 vector (1:9 ratio) using Lipofectamine 2000 transfection reagent (all from Invitrogen). The transfection mix, containing 9 μg of pOG44, 1 μg of the pcDNA5/FRT/TO-based expression vector, 1 ml of Opti-MEM and 20 μL of Lipofectamine 2000, was incubated for 30 min at RT and added directly to Flp-In T-REx-293 Host cells. At 48 h post-transfection, the medium was replaced with DMEM selection medium, additionally supplemented with 100 μg/ml hygromycin B and 5 μg/ml blasticidin S. The medium was replaced every 3 to 4 days to remove dead cells. After 2 to 3 weeks, individual hygromycin-resistant colonies were clonally selected using cloning cylinders (Sigma-Aldrich). The individual colonies were further expanded and stored in liquid nitrogen using the same medium supplemented with 10% DMSO. The expression of recombinant proteins was screened following induction of cells with doxycycline (Sigma-Aldrich) by using immunoblotting and bioluminescence analysis (Supplementary Figure S5).

**Selection of single-cell clones for AGO2 knock-out and AGO2 mutant cell lines**

GFP-positive cells were sorted into 96-well plate (one cell per well) using the BD FACSAria III (BD Biosciences) flow cytometer 48 h post-electroporation. The cells were cultured for about two weeks, after which genomic DNA was isolated with the QuickExtract DNA Extraction Solution (Lucigen Corporation) according to the manufacturer's instructions. Screening of clones harboring deletions (the pair of Cas9_sg1 and Cas9_sg2 caused deletion of 77 bp fragment leading to premature STOP codons) was carried out using GoTaq Polymerase (Promega) according to the manufacturer's instructions with PCR primers AGOe2_F and AGOe2_R. HDR-mediated clones were validated with an assay including PCR with AGOe14_F and AGOe14_R primers and digestion of PCR product using AflII and BseNI restriction enzymes (New England Biolabs). Positive clones (GAC to GCC codon change) should have restriction site for BseNI and lost restriction site for AflII enzyme. Selected clones were sequenced using primer AGOe14_F. Clones with confirmed frame shift or codon change were subjected to western blot analysis. Primer sequences are given in Supplementary Table S8.

**Mycoplasma testing**

All cell cultures used were routinely negatively verified for mycoplasma contamination using Veno GeM Classic Mycoplasma PCR detection Kit (Minerva Biolabs) according to manufacturer's instruction.

**SUPPLEMENTARY TABLES**

**Supplementary Table 1. Publications describing activity of art-miRNAs**

| **Targeted gene** | **Model** | **Type of art-miRNA** | **Reference** (numbers according to main text references) |
| --- | --- | --- | --- |
| *HTT* | human fibroblasts | RNA duplexes | [27] |
| *HTT* | human fibroblasts | RNA duplexes | [28] |
| *ATXN3* | human fibroblasts | RNA duplexes | [33] |
| *HTT* | human fibroblasts, mouse striatal precursor cells | RNA duplexes | [34] |
| *HTT* | human fibroblasts, HD mouse model | chemically-modified single-stranded RNAs | [35] |
| *HTT, ATXN3* | human fibroblasts | chemically-modified RNA duplexes | [36] |
| *HTT* | human fibroblasts | self-duplexing RNAs | [37] |
| *HTT, ATXN3* | human fibroblasts | chemically-modified RNA duplexes | [38] |
| *ATXN3* | human fibroblasts | chemically-modified single-stranded RNAs | [39] |
| *HTT* | human fibroblasts, HD mouse model | chemically-modified single-stranded RNAs | [40] |
| *ATN1* | human fibroblasts | RNA duplexes and chemically-modified RNAs | [29] |
| *HTT*, *ATN1*, *ATXN3* | human fibroblasts, mouse striatal precursor cells | RNA duplexes, self-duplexing RNAs and its chemically modified versions | [30] |
| *ATXN7* | human fibroblasts | RNA duplexes, self-duplexing RNAs and its chemically modified versions | [31] |
| *HTT* | human fibroblasts | self-duplexing RNAs and its chemically modified versions | [32] |

**Supplementary Table 2. Sequences of RNA oligonucleotides.** For art-miRNAs (A2, A4, G2 and G4) nucleotides which form mismatches in the interaction with targeted CAG repeat tract are in bold.

| **Name** | **Sense (5'-3')** | **Antisense (5'-3')** |
| --- | --- | --- |
| A2 | GCUGCUGC**A**GCUGCUGCUGCU | GCUGCUGC**A**GCUGCUGCUGCU |
| siHTT* | GCCUUCGAGUCCCUCAAGUCC | ACUUGAGGGACUCGAAGGCCU |
| siRluc | AUCUGAAGAAGGAGAAAAATT | AUCUGAAGAAGGAGAAAAATT |
| siFluc | CGUACGCGGAAUACUUCGAUU | UCGAAGUAUUCCGCGUACGUU |
| A4 | GCUGCUGC**A**GCUGC**A**GCUGCU | GCUGCUGC**A**GCUGC**A**GCUGCU |
| G2 | GCUGCUGC**G**GCUGCUGCUGCU | GCUGCUGC**G**GCUGCUGCUGCU |
| G4 | GCUGCUGC**G**GCUGC**G**GCUGCU | GCUGCUGC**G**GCUGC**G**GCUGCU |

* from published study, [4].

**Supplementary Table 3. Antibodies**

| **Name (host)** | **Dilution** | **Company (catalogue number)** |
| --- | --- | --- |
| Western blot | | |
| anti-huntingtin (mouse) | 1:1000 | Millipore (MAB2166) |
| anti-atrophin-1 (rabbit) | 1:1000 | Bethyl Laboratories (A300-753A) |
| anti-ataxin-3 (mouse) | 1:1000 | Millipore (MAB5360) |
| anti-GAPDH (mouse) | 1:10000 | Millipore (MAB374) |
| anti-vinculin (rabbit) | 1:1000 | Cell Signaling Technology (4650) |
| anti-plectin (rabbit) | 1:1000 | Abcam (ab83497) |
| anti-firefly luciferase (rabbit) | 1:1000 | Thermo Fisher Scientific (PA5-32208) |
| anti-rabbit HRP-conjugate | 1:2000 | **Jackson ImmunoResearch** (711-035-152) |
| anti-mouse HRP-conjugate | 1:2000 | **Jackson ImmunoResearch** (715-035-150) or Sigma-Aldrich (A9917) |
| Immunocytochemistry | | |
| anti-Pax6 (rabbit) | 1:100 | Cell Signaling Technology (60433) |
| anti-Sox1 (rabbit) | 1:200 | Cell Signaling Technology (4154) |
| anti-Sox2 (rabbit) | 1:200 | Cell Signaling Technology (3579) |
| anti-nestin (mouse) | 1:500 | Stem Cell Technology (60091) |
| anti-rabbit Alexa Fluor 488 | 1:1000 | **Jackson ImmunoResearch** (711-546-152) |
| anti-mouse Alexa Fluor 594 | 1:1000 | **Jackson ImmunoResearch** (715-586-151) |

**Supplementary Table 4. Sequences of DNA oligonucleotides used as PCR primers in RT-qPCR, ddPCR and poly(A) tail length assay.**

| **Name** | **Forward (5'-3')** | **Reverse (5'-3')** |
| --- | --- | --- |
| *GAPDH* | GAAGGTGAAGGTCGGAGTC | GAAGATGGTGATGGGATTTC |
| *Fluc* | AAGAAGTGCTCGTCCTCG | TGATCATGAGCGGCTACG |
| *NlucP* | AAGGTGATCCTGCACTATGGC | TCTTTTTGCCGTCGAACACG |
| *ATXN1* | CAGCCCTGTCCAAACACAAA | GCAACGACCTGAAGATCGAC |
| *ATXN3* | AGTTCAGGAGCACTTGGGAG | CAAAGTGGACCCTATGCTGT |
| *ATXN7* | AGGTGTTCTTAGCGCATCCT | AGTGTGCCATCCATTTTCGG |
| *ATN1* | TGCTATCCATGCAGCCTCTG | AGCAAAGAGCTGGTGACGAA |
| *HTT* | CGACAGCGAGTCAGTGAATG | ACCACTCTGGCTTCACAAGG |
| *PAX6* | TGCTCCGGCATGAAATATACTA | GTCTCCAAATGTGCAGCAAC |
| *SOX1* | ACCAGGCCATGGATGAAG | CTTAATTGCTGGGGAATTGG |
| *NES* | GCGGGCTACTGAAAAGTTCC | CAGGAGGGTCCTGTACGTG |
| *HTT-NlucP - poly(A) length assay* | GCTGTTCCGAGTAACCATCAACG | GCAATAGCATCACAAATTTCACAAATAAAGC |

**Supplementary Table 5. Sequences of oligonucleotides for *ATXN3* smFISH (sequence 5**'**- 3’)**

| 1 | TCGTGGAAGATGGACTCCAT |
| --- | --- |
| 2 | ACAAAGTGAGCCTTCTTGTT |
| 3 | AGTTATTCAGGCAATGTTGA |
| 4 | GGCTAAAATATTCTCCTTGC |
| 5 | CAGCTGATGTGCAATTGAGG |
| 6 | ATCTTCACTAGTAACTCCTC |
| 7 | GCTGCTGTAAAAACGTGCGA |
| 8 | AACCACTGTCATCCATATTT |
| 9 | AGGCATTGCTTATAACCTGA |
| 10 | GGATTAGTTCTAAACCCCAA |
| 11 | CTGATACTCTGGACTGTTGA |
| 12 | TTATAGGATCGATCCTGAGC |
| 13 | CTGTAAACCAGTGTTCCTTA |
| 14 | TTAATTCTGGACCCGTCAAG |
| 15 | GAGCCAAGAAAAGTGCAAGA |
| 16 | AGAATAACCTTCCTGTTGTA |
| 17 | GCAGATCACCCTTAACAACA |
| 18 | AGTTGGTCAGCTTCGCAATC |
| 19 | TTGGACCCTAATCATCTGCA |
| 20 | TAAGTTTTGGTCGATGCATC |
| 21 | AGTTGTGCTAATTCTTCTCC |
| 22 | GTTTTATGGACTCTTTGCTC |
| 23 | CTTCTAACATTCGTTCCAGG |
| 24 | TAACATTCCTGAGCCATCAT |
| 25 | TGCAAATCCTCCTCATCTTC |
| 26 | TGTCAATTTCTTGGCGACTT |
| 27 | AGATCTGCTTCCTCATCTTC |
| 28 | TACTTAGCTGAATAGCCCTG |
| 29 | ATGTTTCTGGAACTACCTTG |
| 30 | TACCTGATGTCTGTGTCATA |
| 31 | TCCGAAGCTCTTCTGAAGTA |
| 32 | GATGTGAACTCTGTCCTGAT |
| 33 | CAAGTGCTCCTGAACTGGTG |
| 34 | ATAGCATCACCTAGATCACT |
| 35 | CTGAAGCATGTCTTCTTCAC |
| 36 | CTAAAGACATGGTCACAGCT |

**Supplementary Table 6. Sequences of DNA oligonucleotides used for cloning for generation of constructs used for transient expression (relative to Figure 2)**

| Name | Forward/sense (5'-3') | Reverse/antisense (5'-3') |
| --- | --- | --- |
| Mutagenesis | | |
| 5'ORF EcoRI | GGCAATCCGGTACTGTTGGTAAA**GAATTC**GCCACCATGGAA | TTCCATGGTGGC**GAATTC**TTTACCAACAGTACCGGATTGCC |
| 5'ORF  EcoR*I*/NdeI | GGTAAA**GAATTC**GCCACCATG**CATATG**GAAGATGCCAAAAACATTAAG | CTTAATGTTTTTGGCATCTTC**CATATG**CATGGTGGC**GAATTC**TTTACC |
| DelA in STOP codon TAA(TA^T) | GCGGCAAGATCGCCGTG**TAT**TCTAGTTGTTTAAAC | GTTTAAACAACTAGA**ATA**CACGGCGATCTTGCCGC |
| Plasmid generation | | |
| ATXN3 3'UTR/3'ORF XbaI/SalI | CGCCATCTACGGGTA**TCTAGA** GAAGAGCTTCGGAAGAGAC | GATCTGACTAATGCT**GTCGAC**TTATCCTGAACTGGTGGCTGGC |
| ATXN3 5’ORF EcoRI/NdeI | GAAATCTACGGGTATC**GAATTC**GCCACCATGGCGGAAGAGCTTCGGAAGAGAC | AGGATCTACGGGTAA**CATATG**TCCTGAACTGGTGGCTGGC |
| HTT 3'UTR/3’ORF XbaI/SalI | GATCATCTACCGTTA**TCTAGA**ATGGCGACCCTGGAAAAGCTG | GATCTGACTAATGCTTT**GTCGAC**TTACAGCGGCGGCGGCTGAGG |
| HTT 5’ORF  EcoRI/NdeI | GAAATCTACGGGTATC**GAATTC**GCCACCATGGCGACCCTGGAAAAGCTG | AGATCTGACTAATGCTTT**CATATG**CAGCGGCGGCGGCTGAGG |
| Synthetic inserts | | |
| CAG17 3’UTR/3’ORF (Xba*I*/Sal*I*) | **CTAGA**ATG(CAG)_17_TAA**G** | **TCGAC**TTA(CTG)_17_CAT**T** |
| CAG17 5’ORF (EcoR*I*/Nde*I*) | **AATTC**GCCACCATGGAG(CAG)_17_**CA** | **TATG**(CTG)_17_C TCCATGGTGGC**G** |

**Supplementary Table 7. Sequences of DNA oligonucleotides used for cloning for generation of constructs used for stable expression (relative to Figure 4)**

| **ID** | **Oligo Name** | **Sequence (5'-3')** |
| --- | --- | --- |
| **1** | Fluc_KpnI_F | GCGGTACCGCCACCATGGAAGATGCCAAAAACATTAAG |
| **2** | Fluc_BamHI_R | CGGGATCCTCATTACACGGCGATCTTGCCGCCCTTCTTG |
| **3** | SV40pA_BglII_F | GAAGATCTCAGACATGATAAGATACATTGATG |
| **4** | SV40pA_BamHI_R | ATGTGCGGCCGCGGATCCTACCACATTTGTAGAGGTTTTACTTGC |
| **5** | BI16_F1_XbaI_F | GCTCTAGAGTACATCAAGTGTATCAT |
| **6** | BI16_F1_KpnI_R | GGCGGTACCGACGATCTCTATCACTG |
| **7** | BI16_F2_XbaI_F | GCTCTAGAAAATGTCGTAACAACT |
| **8** | BI16_F2_BamHI_R | GGCGGATCCGACGATCTCTATCACTG |
| **9** | SV40pA_BclI_F | GCGCTGATCACAGACATGATAAGATACATTGATG |
| **10** | SV40pA_SphI_R | CCGTGCATGCTACCACATTTGTAGAGGTTTTACTTGC |
| **11** | NlucP_XhoI_F | CCGCTCGAGATGGTCTTCACACTCGAAGATTTCG |
| **12** | NlucP_ApaI_R | CTGGGCCCTCATTAGACGTTGATGCGAGCTGAAGCAC |
| **13** | Linker 13/14 ss | (Phosp)TCGAGCATCATCACCACCATCATGAGAACCTGTACTTTCAGAGCGATTACAAGGATGACGACGATAAGT |
| **14** | Linker 13/14 as | (Phosp)TCGAACTTATCGTCGTCATCCTTGTAATCGCTCTGAAAGTACAGGTTCTCATGATGGTGGTGATGATGC |
| **15** | ATGLinker 15/16 ss | (Phosp)GATCCGCCACCATGGATTACAAGGATGACGACGATAAGC |
| **16** | ATGLinker 15/16 as | (Phosp)TCGAGCTTATCGTCGTCATCCTTGTAATCCATGGTGGCG |
| **17** | HTT_BamHI_F | CGGGATCCGCCACCATGGCGACCCTGGAAAAGCTGATG |
| **18** | HTT_XhoI_R | GCATCTCGAGTGGTCGGTGCAGCGGCTCCTCAGC |
| **19** | AGO2_HindIII_F | CCCAAGCTTGCCGCCATGGACTACAAGGACG |
| **20** | AGO2_BamHI_R | GGCGGATCCTTATCAAGCAAAGTACATGGTGCGCAG |

**Supplementary Table 8. Sequences of DNA oligonucleotides used for sgRNA constructs and validation of genomic DNA editing**

| **ID** | **Sequence 5’- 3’** | **Description** |
| --- | --- | --- |
| sgRNA1s | CACCGAGGCTTGAAGGCATATCCT | oligo for Cas9_sg1 plasmid construction |
| sgRNA1a | AAACAGGATATGCCTTCAAGCCTC | oligo for Cas9_sg1 plasmid construction |
| sgRNA2s | CACCGACAGGCCAATTTCTTCGAAA | oligo for Cas9_sg2 plasmid construction |
| sgRNA2a | AAACTTTCGAAGAAATTGGCCTGTC | oligo for Cas9_sg2 plasmid construction |
| sgRNA3s | CACCGTCTGCTCCCAGAAAGATGA | oligo for Cas9_sg3 plasmid construction |
| sgRNA3a | AAACTCATCTTTCTGGGAGCAGAC | oligo for Cas9_sg3 plasmid construction |
| AGOe2_F | AATGCAATATTGGCCTGGAC | forward primer for exon2 of *Ago2* gene |
| AGOe2_R | CGCAGACCACTTACACAGGT | reverse primer for exon2 of *Ago2* gene |
| AGOe14_F | CACCTCCAAAAGGTGATGGT | forward primer for exon2 of *Ago2* gene |
| AGOe14_R | AGCTTAGTGAGACCCCATGC | reverse primer for exon2 of *Ago2* gene |
| U6-Fwd | GAGGGCCTATTTCCCATGATTCC | sequencing primer for sgRNA validation |
| ssODN | GACGCCACTCCTCTCCGCAGGCCGCCGGTGTTCCAGCAGCCAGTCATCTTTCTGGGAGCAGCCGTCACTCACCCCCCCGCCGGGGATGGGAAGAAGCCCTCCATTGCCGCCGTGAGTGTCAGC | donor template for HDR |

**SUPPLEMENTARY REFERENCES**

1. Chang T-H, Huang H-Y, Hsu JB-K, et al (2013) An enhanced computational platform for investigating the roles of regulatory RNA and for identifying functional RNA motifs. BMC Bioinformatics 14 Suppl 2:S4.

2. Gu S, Jin L, Zhang F, et al (2009) The biological basis for microRNA target restriction to the 3’ untranslated region in mammalian mRNAs. Nat Struct Mol Biol 16:144–150.

3. Sammarco MC, Grabczyk E (2005) A series of bidirectional tetracycline-inducible promoters provides coordinated protein expression. Anal Biochem 346:210–6.

4. Wang YL, Liu W, Wada E, et al (2005) Clinico-pathological rescue of a model mouse of Huntington’s disease by siRNA. Neurosci Res 53:241–249

**SUPPLEMENTARY FIGURES**

**
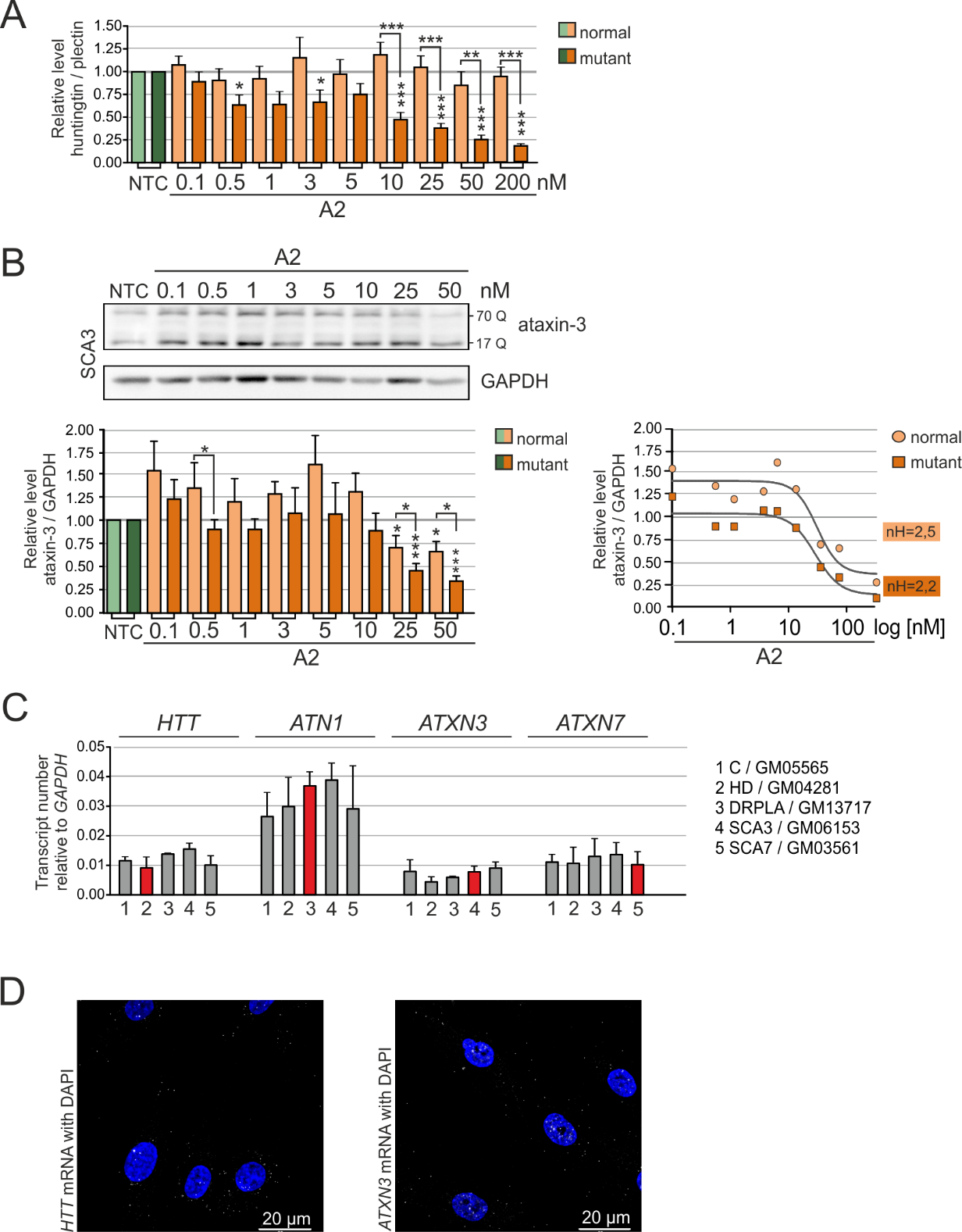
**

**Supplementary Figure 1. Additional data for Figure 1**

**(A)** Detailed results corresponding to western blot analysis of huntingtin levels in HD-patient derived fibroblasts presented in Fig. 1C. NTC – cells treated with non-targeting siRNA (BlockIT siRNA). n=3 **(B)** Western blot analysis of ataxin-3 levels in SCA3 fibroblasts lysed 72 h after transfection with indicated concentration of A2. Results are presented as dose-response curves which were used for indicated Hill’s coefficient calculation. NTC – cells treated with non-targeting siRNA (BlockIT siRNA). For (A) and (B) data were analyzed using one-way ANOVA (with Bonferroni multiple comparisons test) n=3 **(C)** ddPCR-based quantification of indicated transcripts number in indicated fibroblast cell lines (disease and repository number are given). Two isolations of total RNA were performed from independent cultures of fibroblasts to obtain two sets of cDNA for this analysis. Red bars represent result obtained in cell line with mutant transcript. **(D)** Representative smFISH images for *HTT* (left panel) and *ATXN3* (right panel) mRNAs in healthy person-derived fibroblasts. DAPI was used for nuclear staining.


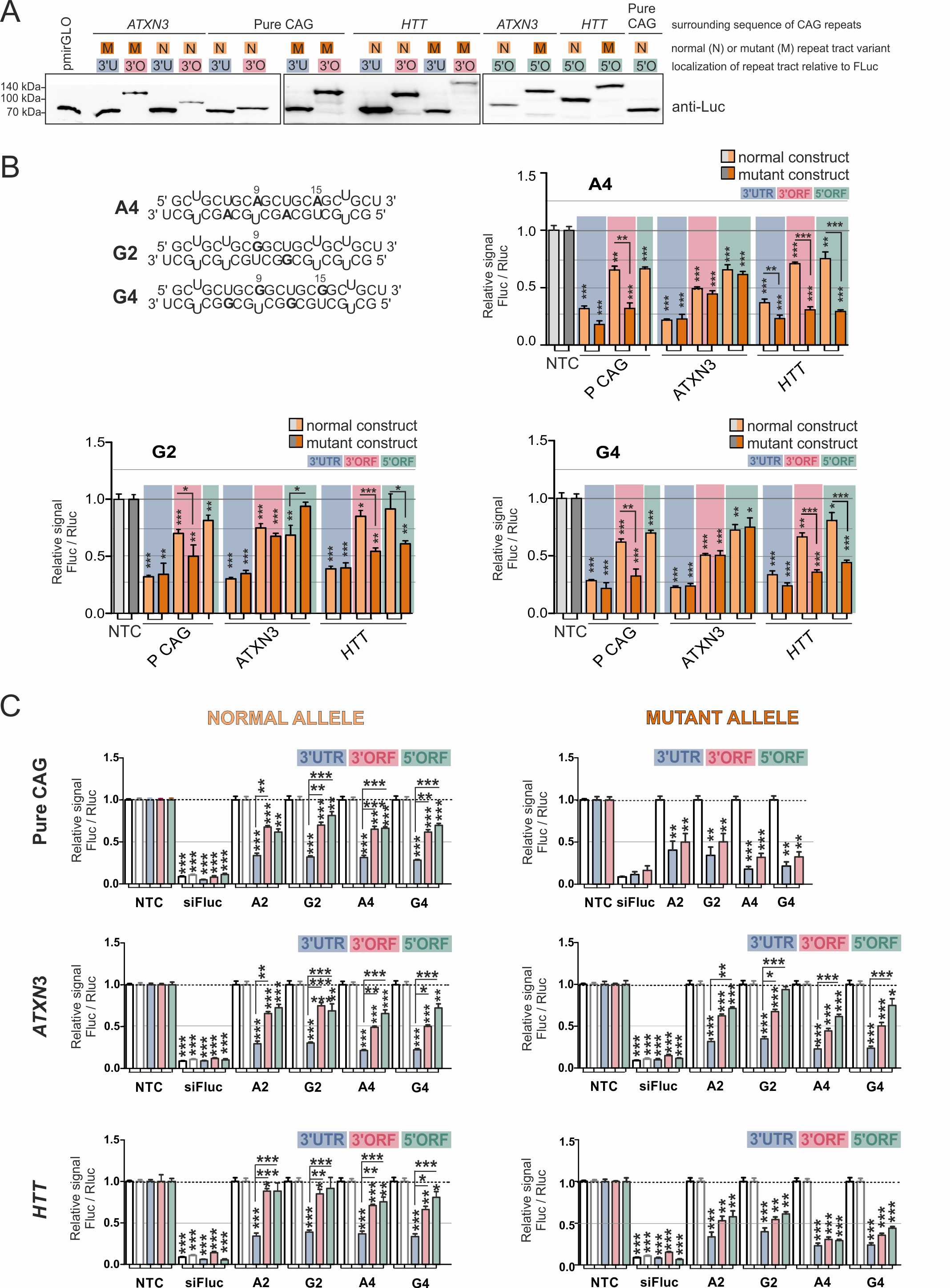


**Supplementary Figure 2. Additional data for Figure 2**

**(A)** Western blot for Fluc, expressed in HEK 293T cells transfected with designed pmirGLO-based constructs. Fusion proteins include addition of normal or mutant CAG repeat tract length, various location of repeat tract (3'U - 3'UTR, 3'O - 3' site of ORF, 5'O - 5' site of ORF), as well as different repeat tract surrounding sequence. **(B)** Sequences of A4, G2 and G4 art-miRNAs and predicted base-pairing of two strands within a duplexes. Luciferase assay performed 24 h after cotransfection of HEK 293T cells with 50 nM A4, G2 or G4 and 100 ng of indicated plasmids. C – light grey: cells treated with pmirGLO plasmid and non-targeting siRNA (NTC), dark grey: “5’ORF”-modified pmirGLO plasmid treated with non-targeting siRNA (signal normalization details are given in Materials and Methods). Data was analyzed using one-way ANOVA (with Bonferroni multiple comparisons test). n=3 **(C)** Different representation of data shown in Fig. 2C and panel (B) of this figure. Graphs present activity of a set of art-miRNAs (A2, G2, A4 and G4), together with results for siRNA targeting *Fluc*, for selected construct: with normal repeat tract (left panels) or mutant one (right panels) and with various repeat tract surrounding sequence (Pure CAG - no additional sequence added or from *HTT* or *ATXN3* genes). n=3


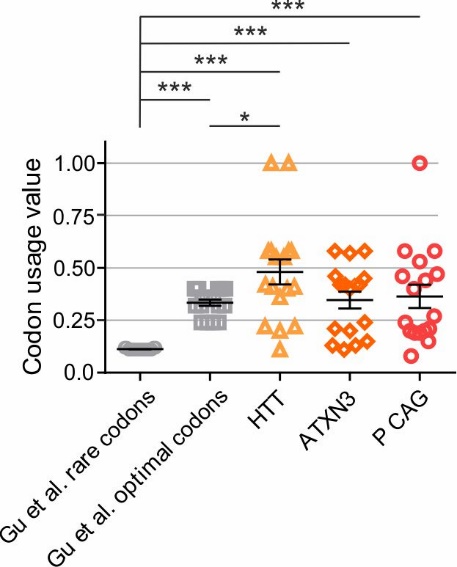


**Supplementary Figure 3. Codon usage bioinformatic analysis**

Codon usage of 17 codons upstream to CAG repeat tract in *HTT, ATXN3* and in 3’ORF *Fluc* (upstream to MCS and STOP codon of pmirGLO). As controls we used codon described as rare or optimal in Gu et al., 2009. Data was analyzed using one-way ANOVA (Bonferroni multiple comparisons test).


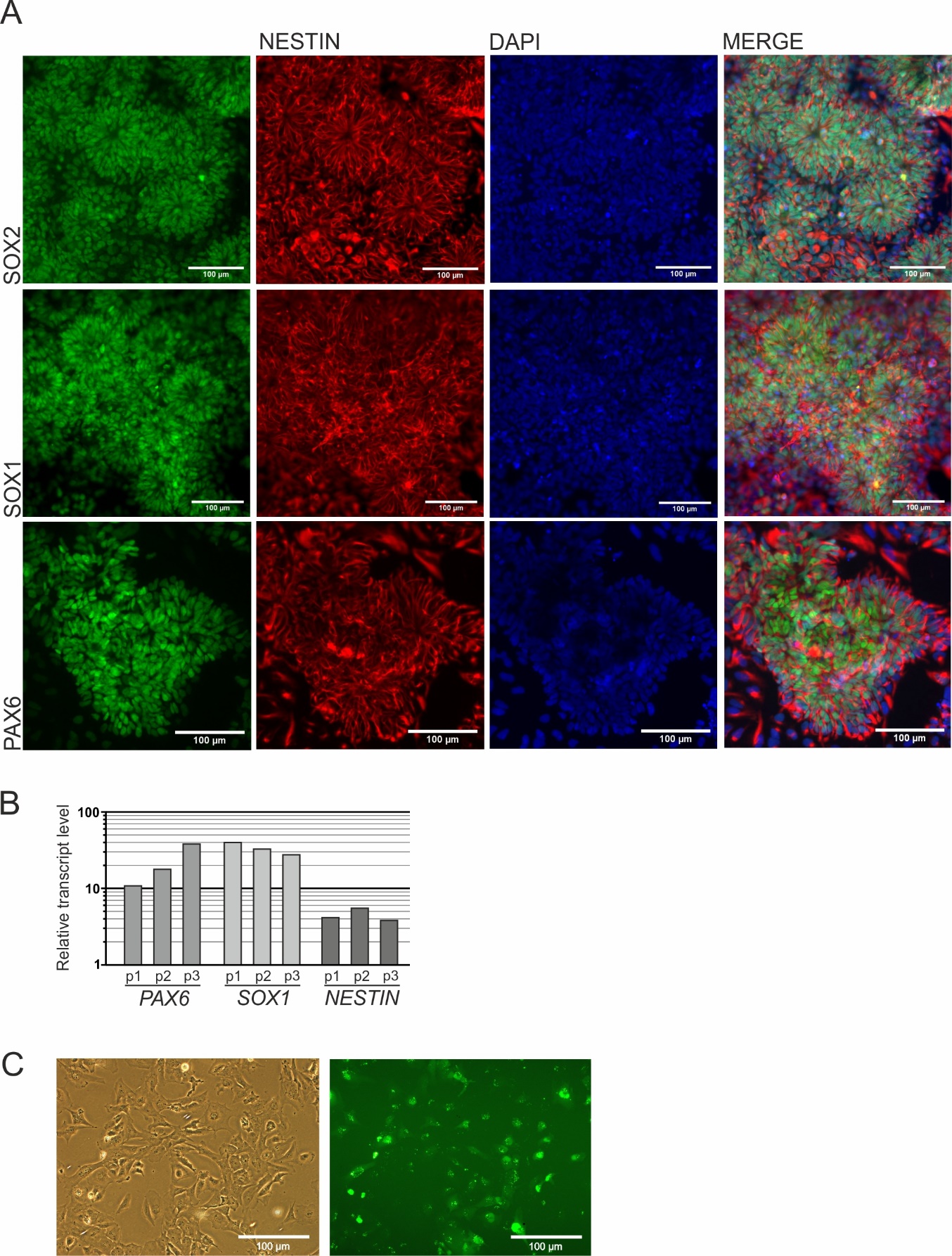


**Supplementary Figure 4. Additional data for Figure 3**

**(A)** Immunofluorescence staining of HD NPs for selected markers for neural stem cells. **(B)** RT-qPCR results of expression level for selected neural stem cells markers: *PAX6*, *SOX1* and *NESTIN* in HD NPs, analyzed at passage 1, 2 and 3 (indicated as p1, p2 and p3). Results are presented as mean relative to expression level in parental HD iPSCs, set at 1, and were normalized to GAPDH expression level. **(C)** Exemplary images of HD NPs 24 h after transfection of 100 nM BlockIT with siPORT Amine.


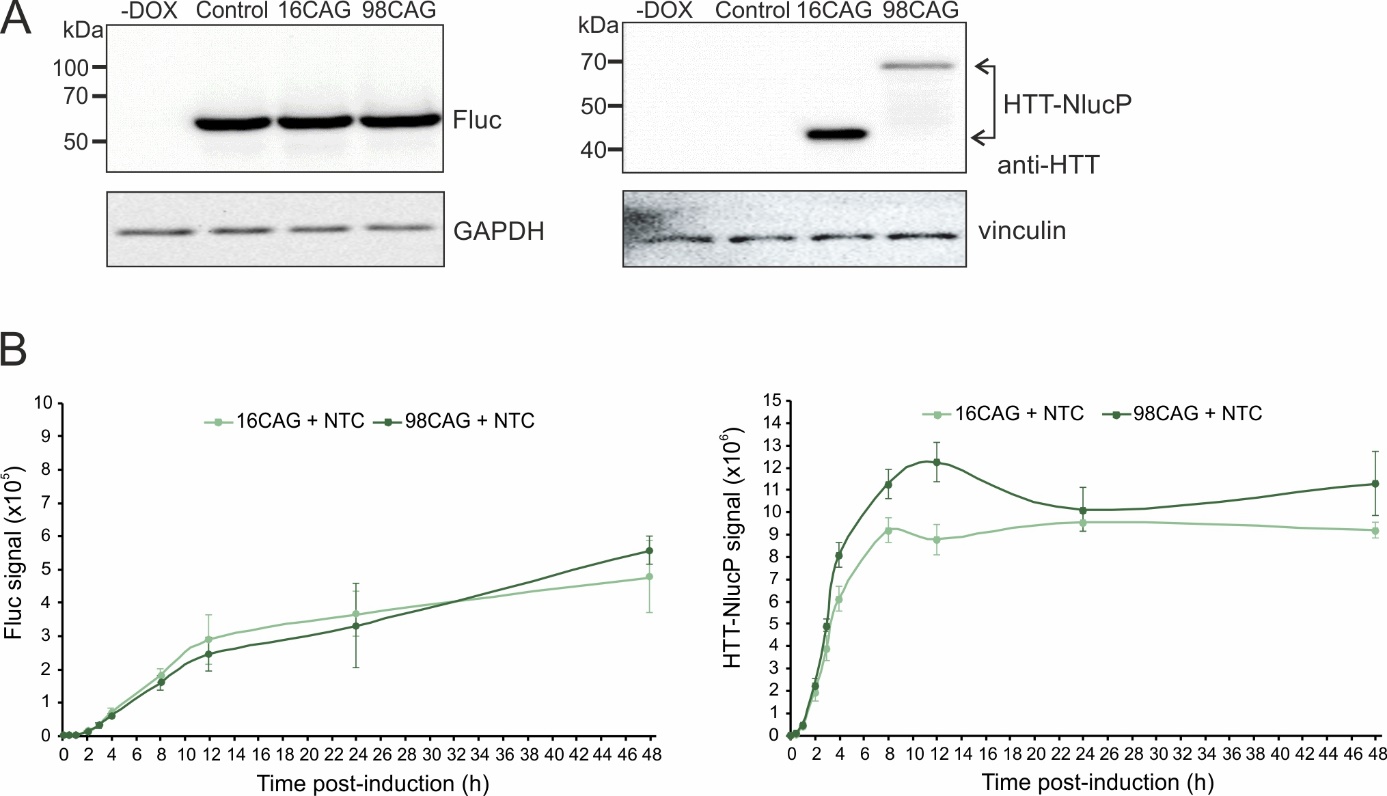


**Supplementary Figure 5. Additional data for Figure 4: Flp-In T-REx-293 cell lines**

**(A)** Western blot for Fluc (left panel) and HTT-NlucP fusion protein in generated 16CAG and 98CAG Flp-In T-REx-293 cell lines, as well as control cell line (lacking CAG repeat insert). Control, 16CAG and 98CAG lysates were prepared 24 h after induction with doxycycline. GAPDH or vinculin were used as protein loading controls. **(B)** Time course of Fluc (left panel) and HTT-NlucP (right panel) signal after induction. 16CAG and 98CAG cell lines were transfected with control siRNA (NTC, siRluc) and 12 h later expression was induced with doxycycline. Cells were harvested at 30 min, 1, 2, 3, 4, 8, 12, 24 and 48 h points, counted and the same number of cells were lysed and processed for Nano-Glo Dual-Luciferase Reporter Assay. Luciferase activity is given in arbitrary units. n=3


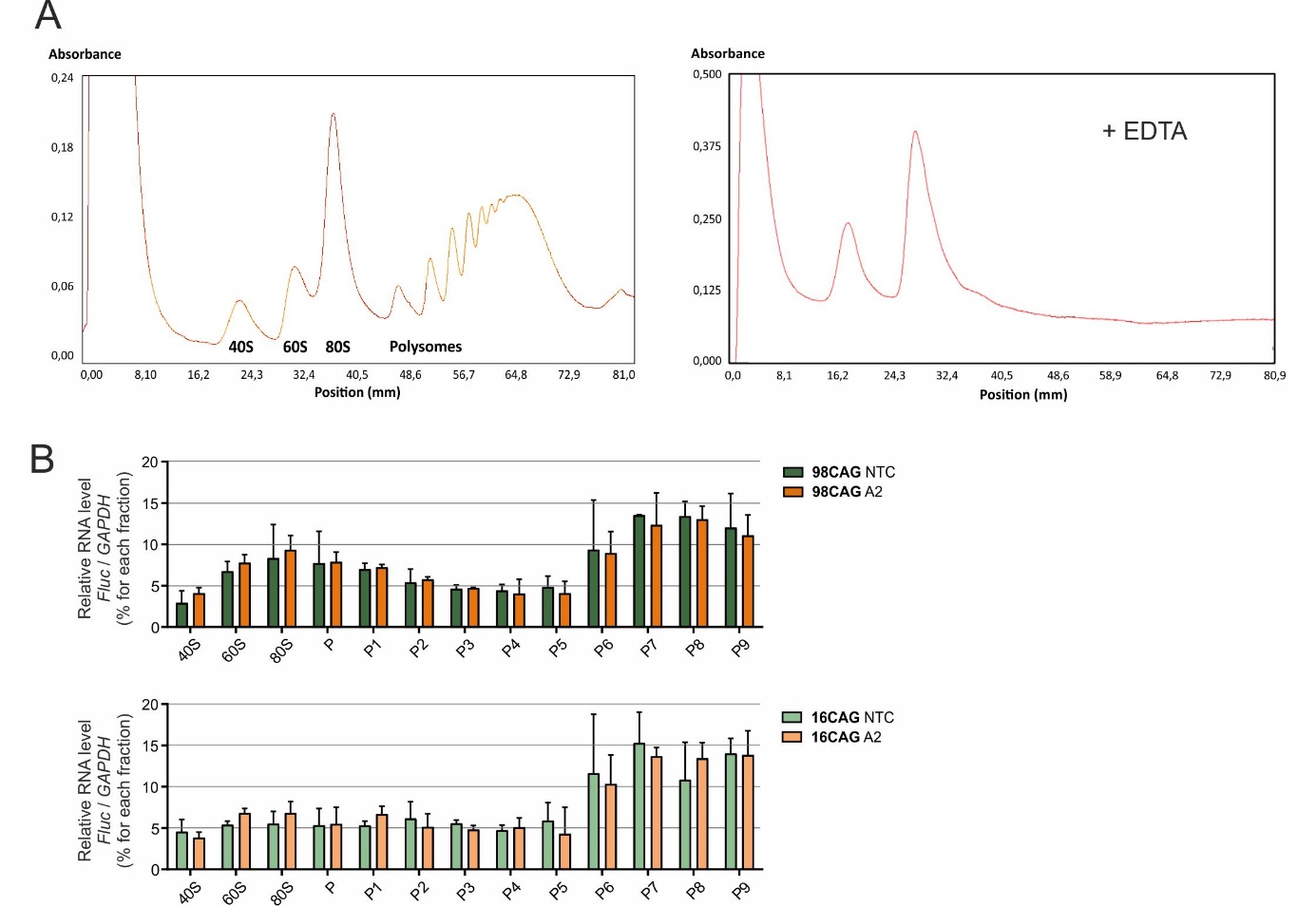


**Supplementary Figure 6. Additional data for Figure 4: polysome profiling**

**(A**) Left panel: exemplary graph of A260 profile obtained in sucrose gradient separation of Flp-In T-REx-293 cell line lysate, right panel: analogous results obtained after addition of 20 nM of EDTA to lysis buffer and sucrose solutions. **(B)** RT-qPCR results of control *Fluc* expression level after transfection of 98CAG (upper panels) or 16 CAG (lower panels) cell line with 100 nM A2 or control siRNA (NTC, siRLuc), at 3 h time point after induction, in indicated fractions of ribosomal subunits (40S and 60S), 80S monosome and polysomes (P-P9). Data were normalized to *GAPDH* expression in each fraction and are presented as % share of *HTT-NlucP* expression where 100 % is the sum of obtained values for all fractions. Data analysis using two-way ANOVA showed lack of statistically significant results in comparison of *Fluc* expression after treatment. n=3

**
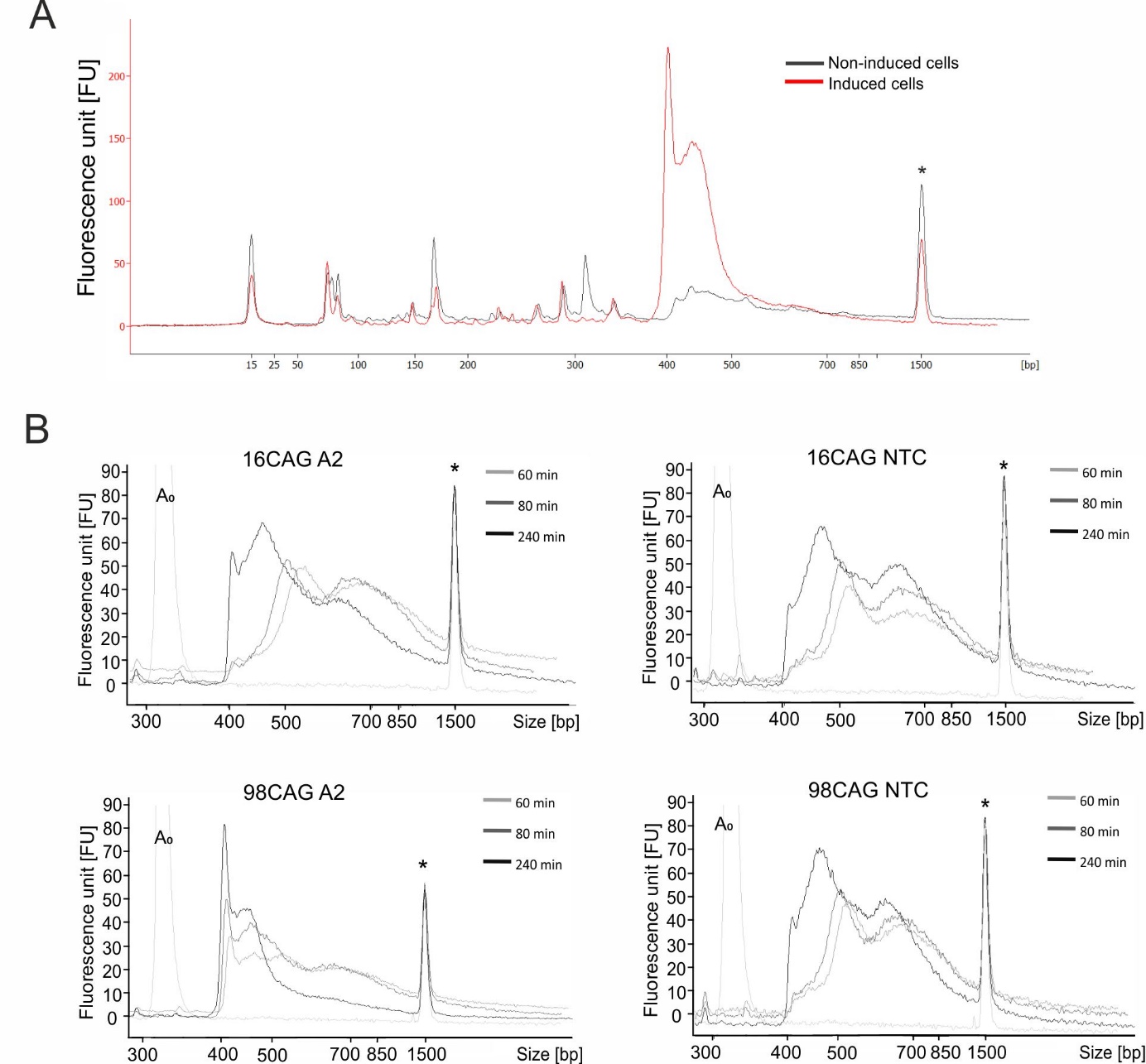
**

**Supplementary Figure 7. Additional data for Figure 4: poly(A) tail-length assay**

**(A)** Example of raw data for resolution of products obtained from poly(A) tail length assay for 98CAG cell line: non-induced cells and cells 4 h after induction and treatment with A2. **(B)** Different graphs containing data from the Figure 4F for clear presentation of time-course changes in representation of poly(A) tails for specific cell line and after specific siRNA treatment: analysis of poly(A) tails lengths of *HTT-NlucP* transcript in 98CAG cells at indicated time points (60, 80, 120 min) after transfection with 100 nM A2 or control siRNA (NTC, siRLuc). Estimated poly(A) tails lengths are indicated. Experiment was repeated and similar results were obtained. A_0_ - peak obtained with reporter-specific primers amplifying a region upstream of the polyadenylation site. * - an internal standard peak (1500 bp Upper Marker).


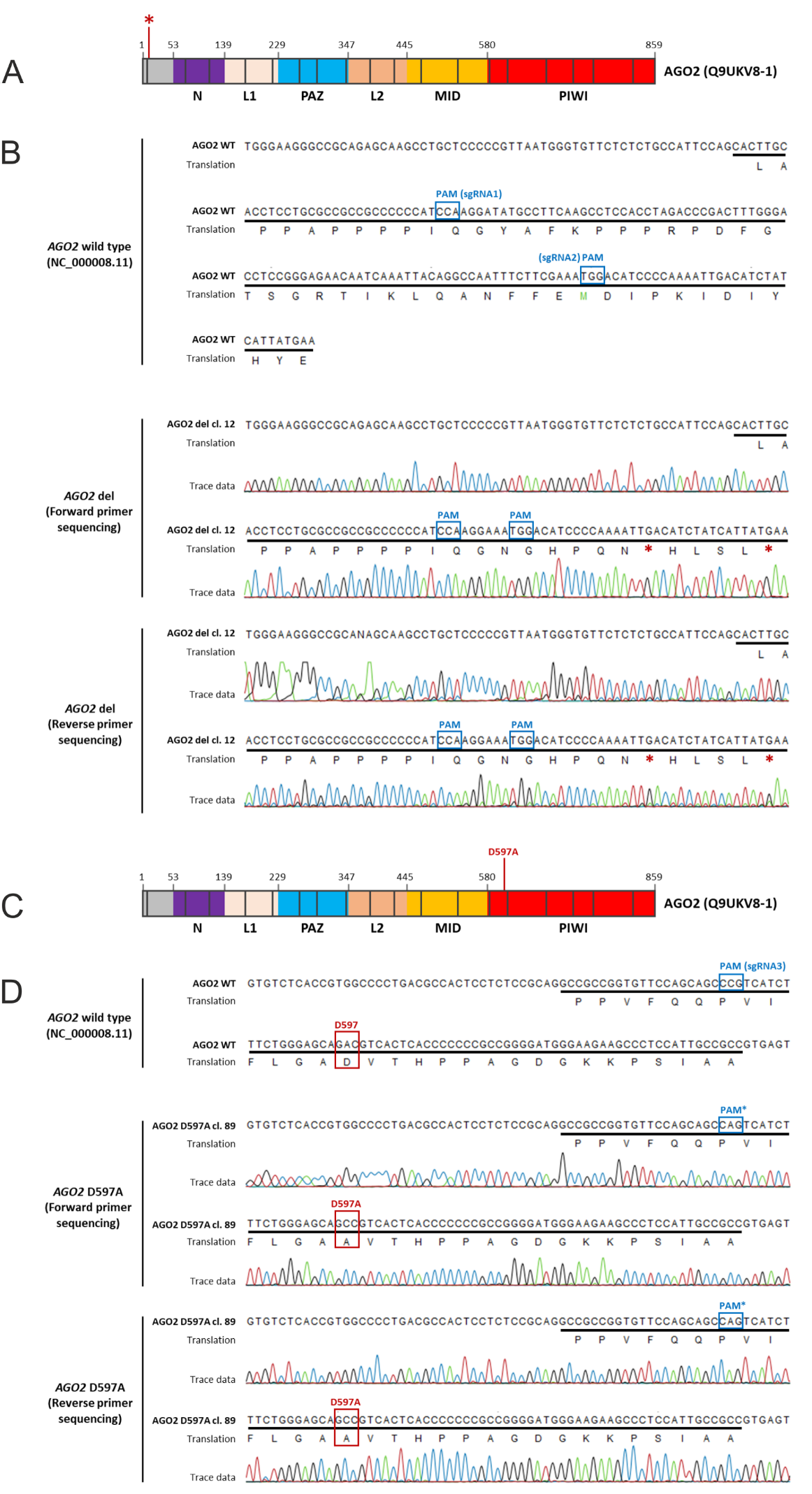


**Supplementary Figure 8. Additional data for Figure 5. AGO2 knockout and mutant cell lines**

**(A)** and **(C)** Schematic architecture of AGO2 protein (Q9UKV8-1) with characteristic protein domains (N, L1, PAZ, L2, MID, PIWI). The positions of CRISPR/Cas9-mediated knockout **(A)** and D597A mutation **(C)** are indicated by red lines. **(B)** DNA sequencing confirmation of correct homozygous AGO2 knockout in 98CAG Flp-In T-REX-293 cell line (AGO2del). Wild type *AGO2* reference sequence (NC_000008.11), as well as, chromatograms from forward and reverse primer DNA sequencing obtained from genomic DNA isolated from AGO2del clone 12 are shown. Underlined DNA sequence corresponds to exon 2. PAM sequences for sgRNA1 and sgRNA2 are indicated by blue frames. As a consequence of CRISPR/Cas-9 mediated knockout a deletion of 77 bp fragment is observed leading to the generation of premature termination codons indicated by asterisks. **(D)** DNA sequencing confirmation of correct homozygous AGO2(D597A) mutant 98CAG Flp-In T-REX-293 cell line (AGO2mut). Underlined DNA sequence corresponds to exon 14. Wild type *AGO2* reference sequence (NC_000008.11), as well as, chromatograms from forward and reverse primer DNA sequencing obtained from genomic DNA isolated from AGO2mut clone 89 are shown. PAM sequence for sgRNA3 is indicated by blue frame. As a consequence of CRISPR/Cas-9 mediated HDR-depended insertion D597A mutation (GAC to GCC) is observed, indicated by red frame. To prevent CRISPR/Cas-9 mediated re-cleavage a silent mutation in the PAM sequence was introduced (PAM*).
